# Plant Taxa as Raw Material in Plant-Based Meat Analogues (PBMAs)—A Patent Survey

**DOI:** 10.3390/nu16234054

**Published:** 2024-11-26

**Authors:** Kinga Kostrakiewicz-Gierałt

**Affiliations:** Department of Tourism Geography and Ecology, Institute of Tourism, Faculty of Tourism and Recreation, University of Physical Education in Kraków, Jana Pawła II 78, 31-571 Krakow, Poland; kinga.kostrakiewicz@awf.krakow.pl

**Keywords:** meat analogue, nutritive value, patent, PRISMA, useful plant, underutilized plant

## Abstract

**Background/Objectives**: The environmental problems associated with meat production, the pain and distress of animals, and health problems have contributed to the increased appreciation of meat alternatives. **Methods**: The review of patented inventions presenting plant-based meat analogues (PBMAs) issued in the years 2014–2023 was conducted according to PRISMA statements across the ISI Web of Science, as well as Google Patents and Espacenet Patent Search engines. **Results**: The analysis of 183 patents showed an increase in patent numbers in the years 2020–2022, with the greatest number of patents developed by teams consisting of two authors. The authors and patent applicants were mainly affiliated with the United States, while Société des Produits Nestlé S.A. emerged as the leader among applicant institutions. The International Patent Classification (IPC) codes were given to 177 patents, which were mainly classified as Human Necessities (Section A). In total, inventors mentioned 184 taxa, including 28 genera, 1 section, 144 species, 5 subspecies and 6 varieties of vascular plants. The majority of taxa showed a high edibility rating and belonged to perennials, mainly herbaceous plants representing the families Fabaceae, Poaceae and Brassicaceae. The analysis of patents showed that plants are a promising source of protein, lipids, fibre, polyphenols, starch and gum in meat analogues. At the same time, the noticed slight use of numerous taxa, despite the substantial content of valuable constituents as well as high edibility rates, presumably might be caused by their underutilization in numerous regions of the world. **Conclusions**: The direction of future studies should focus on searching for novel plant-based meat analogues based on underutilized, promising plant sources and investigations of their usefulness.

## 1. Introduction

Meat consumption is rising annually as human populations grow and affluence increases [1]. However, the environmental problems associated with meat production, such as the greenhouse effect, overuse of land resources, the pain and distress of animals, as well as health problems, e.g., cardiovascular and intestinal diseases, have contributed to the increased appreciation of meat alternatives. These encompass four primary categories based on their origin: plant-based, microorganism-based, insect-based, and lab-grown animal cell-based meat analogues [2,3,4,5], attracting the attention of consumers to varying extent, often depending on the country of origin and residence [6]. The voluminous literature [7,8,9,10,11,12,13,14,15,16,17,18] evidences that plant-based meat alternatives (PBMA’s), i.e., products made from plant-derived materials that mimic the appearance, flavour, mouth feel, fibrous texture, and chemical characteristics of meat, have for a long time attracted the attention of numerous scientists and are rising stars of the food industry. The consumption of plant-based meat alternatives (PBMAs) containing highly beneficial essential amino acids, low saturated fat, and being cholesterol-free is associated with numerous health benefits. Several researchers reported that plant-based meat has various health advantages, such as reducing obesity-induced metabolic dysfunction, cardiovascular disease, strokes, and cancer [7,8,16]. Also, the consumption of plant-based meat alternatives (PBMA’s) contributes to anti-inflammation and immune activity [7,8]. Moreover, the improvement of clinical indices in type 2 diabetes contributes to weight loss and weight maintenance, while the consumption of meat analogues that are supplemented with dietary fibre helps in the improvement of gut health [7,8]. On the other hand, Ishaq et al. [12] argued that the proper understanding of the mechanism of the gastrointestinal fate of plant-based meat analogues is very important, and it might allow researchers to obtain better knowledge about the digestibility and bioavailability of meat analogues. Simultaneously, He et al. [7] reported that the transition from heavy meat to plant-based diets might contribute to an estimated 6% to 10% reduction inthe global mortality rate.

The production of plant-based meat alternatives (PBMA’s) is not a new food category (e.g., [7,8,12,16]). The aforementioned authors claim that the perception of plant-based meat alternatives as a source of protein has occurred since ancient times, comprising traditional plant-based meat analogues like tempeh, seitan and tofu. In the early 20th century, cereal-based and nut products (e.g., Nuttose and Protose) appeared. After the Second World War, extruded wheat gluten, soy protein concentrates, and defatted soy meal helped to expand the concept of texturized vegetable proteins. In 1980, Tofurky and other similar products were developed to target the vegetarian demographic niche. During the early 21st century, with the help of modern technologies and developments in food engineering, plant-based meat has mimicked the appearance, taste, texture, and functional properties of sausages, fillets and burgers. Currently (2015–2023), the worldwide development of the plant-based meat alternative marketplace is growing with rapid progress in availability and product offering. As stated by Boukid [9], the prevailing health consciousness, as well as concern for animal welfare and the future environment, has lifted the concept of plant meat alternatives from niche to the mainstream, and the market for plant-based meat analogues in North America and Europe has extended beyond just vegan, vegetarian or flexitarian customers to meat-eating and meat-loving customers. The promising results of survey questionnaires [19,20,21,22,23,24], as well as sensory evaluations [25,26] conducted in numerous European, American and Asian countries, allow one to expect that plant-based meat alternatives have the best chance of successfully replacing meat when they closely resemble highly processed meat products in taste and texture and are offered at competitive prices.

To date, numerous original papers (e.g., [27,28,29]) and academic reviews (e.g., [5,8,10,12,30,31,32,33,34]) have appeared, focusing on the use, physical-chemical properties and functionality of plant species as ingredients in plant-based meat analogues. The aforementioned authors focused on investigations of proteins, fats, stabilizing agents, colourants and flavourings deriving from the most common plant sources, such as legumes (soy, pea, chickpea, lentils, beans, peanuts), cereals and pseudocereals (wheat, corn, oats, quinoa, amaranth, buckwheat), as well as oil seeds (hemp, sunflower, rapeseed, sesame). Nevertheless, taking into account the number of edible plants, it might be stated that despite growing interest in the role of plants as raw material in meat substitutes, the current state of knowledge is still insufficient. The deficiency of publications considering the use of plant taxa in patented meat substitutes seems to be particularly noticeable. Considering this, the present investigation concentrated on the analysis of patents presenting plant-based meat analogues (PBMAs). The specific aims of the performed investigation focused on the characteristics of (i) patented inventions regarding the number and country affiliation of authors and applicants, International Patent Classification (ICP) codes, as well as the number of forward citations; (ii) plant taxa used in patented plant-based meat analogues (PBMAs) regarding their taxonomic affiliation, life form, lifespan, edibility rate, as well as use as a raw material.

## 2. Materials and Methods

### 2.1. Patent Search

Patents were searched by browsing the ISI Web of Science (all Databases), the most widely used for bibliometric analyses, as well as Google Patents and Espacenet Patent Search engines, gathering the largest number of open access patents [35,36]. The survey of literature records published from 1 January 2014 up to 31 December 2023 was carried out according to PRISMA statements [37] with factorial combinations of the following keywords in the searches: (‘plant’) and (’meat analogue’ or ‘meat alternative’ or ‘meat substitute’).

The selection terms were observed in the title and Abstract. The patent search was conducted from 1 June to 30 July 2024. Due to the number of records, ranging from a dozen to tens of thousands after texting particular combinations of keywords, the analysis was limited to the first 300 records. Such a number was estimated as appropriate after a pilot study showing that with an increasing record number of over 300, the number of duplicates has augmented substantially. Therefore, the patent search included 900 hits from the ISI Web of Science, 900 from Google Patents, and 900 from Espacenet. Following the removal of duplicates (publications indexed in more than one database), the Abstracts and Descriptions of patents were screened for relevance and eligibility.

### 2.2. Study Eligibility and Selection

**Figure 1:**
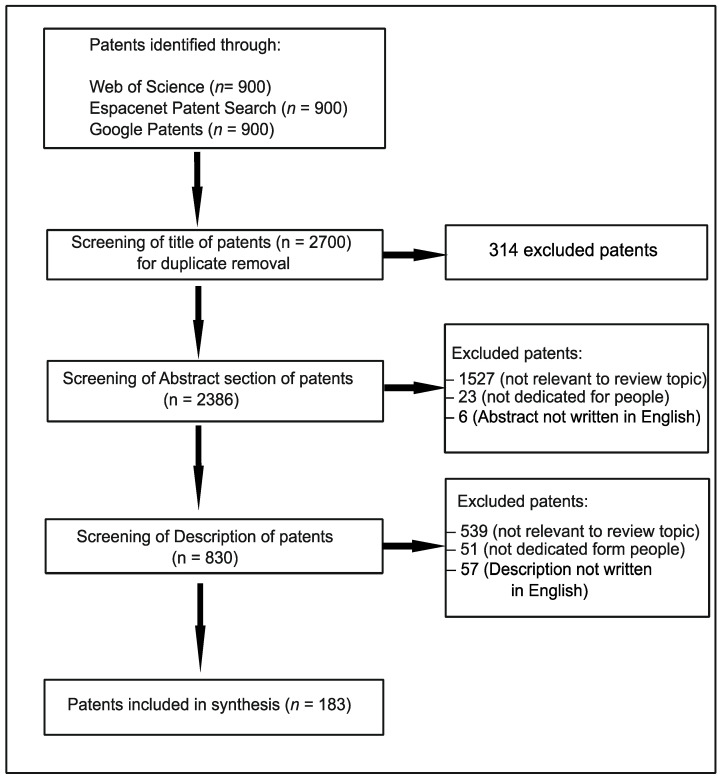
The procedure for the patent search.

During the screening of the Abstract and Description of patents, the inclusion criteria were as follows: (i) the invention presents a plant-based meat analogue, (ii) the description of the invention contains a specification of plant taxa used as a source of particular constituents in meat substitutes, (iii) the plant-based meat analogue is suitable for humans and (iv) the Abstract and Description of the patent were written in English. The exclusion criteria were as follows: (i) the invention is not relevant to the main topic of review (e.g., refers to methods of modification of meat analogue flavour and taste, presents methods and apparatus useful in meat substitute freshness or water content detection, describes devices useful in the production of meat substitutes), (ii) the basis of meat analogue are taxa not belonging to the plant kingdom (e.g., fungi), (iii) the invention is dedicated to animals, (iv) the Abstract and patent Description are not written in English. A chart detailing the patent search procedure is presented in Figure 1.

To assess the quality of the included studies and reduce the potential for misclassification, the Abstracts and then Description of patents were subjected to a critical double screening. From eligible patents, the following data were extracted: author names, author number, affiliation of first author, year of publication, title of patent, patent applicant (institution or individual person filing the patent application) name and affiliation, International Patent Classification (IPC) code according to Anonymous [38] listed as first (in the case of more than one code), number of forward citations (citations by authors of later patents), plant taxa as sources of particular constituents. The aforementioned data were extracted using a form created in Microsoft Excel 2007.

### 2.3. Statistical Analysis

The statistical significance of differences in the number of inventions (i) developed by different numbers of authors, (ii) with a different country affiliation of the first author, and (iii) with a different country affiliation of applicants was checked using the non-parametric Kruskal–Wallis H test. The statistical significance of the correlation between the year of patent publication and the number of citations was tested by applying the Pearson coefficient (at the level < 0.05).

## 3. Results

### 3.1. Patent Analysis

**Figure 2:**
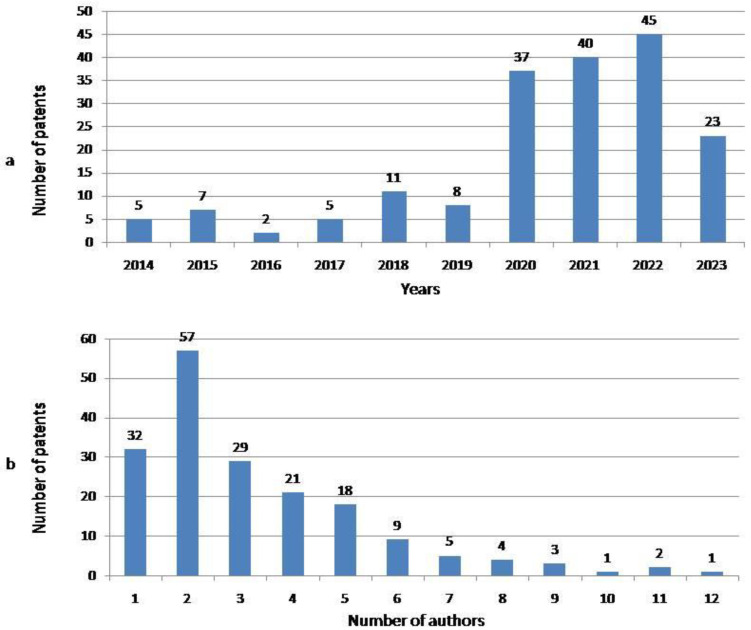
The number of patents referring to plant-based meat analogues (PBMA) published in particular years of the period 2014–2023 (**a**) and developed by different numbers of authors (**b**).

During the study period, altogether 183 patents [39,40,41,42,43,44,45,46,47,48,49,50,51,52,53,54,55,56,57,58,59,60,61,62,63,64,65,66,67,68,69,70,71,72,73,74,75,76,77,78,79,80,81,82,83,84,85,86,87,88,89,90,91,92,93,94,95,96,97,98,99,100,101,102,103,104,105,106,107,108,109,110,111,112,113,114,115,116,117,118,119,120,121,122,123,124,125,126,127,128,129,130,131,132,133,134,135,136,137,138,139,140,141,142,143,144,145,146,147,148,149,150,151,152,153,154,155,156,157,158,159,160,161,162,163,164,165,166,167,168,169,170,171,172,173,174,175,176,177,178,179,180,181,182,183,184,185,186,187,188,189,190,191,192,193,194,195,196,197,198,199,200,201,202,203,204,205,206,207,208,209,210,211,212,213,214,215,216,217,218,219,220,221] referring to plant-based meat analogues (PBMA) were recorded (Table A1). The number of patents per year ranged from 2 in the year 2016 to 45 in 2022 (Figure 2a). The number of authors amounted from 1 to 12 (Figure 2b). The greatest number of inventions was developed by a research team consisting of 2 authors, whereas the lowest number of patents was developed by research teams consisting of 10 to 12 people. The Kruskal–Wallis H test (H = 48.76; *p* < 0.001) confirmed that the number of patents invented by teams of two scientists in particular years was significantly greater than by teams consisting of more than eight authors (Table A2). The number of forward patent citations ranged from 0 to 100 (Table 1). The majority of patents were not cited. Among cited patents, the majority were cited once. The Pearson coefficient (r = −0.50) showed the occurrence of a statistically significant negative correlation between the year of patent publication and the number of its citations.

**Figure 3:**
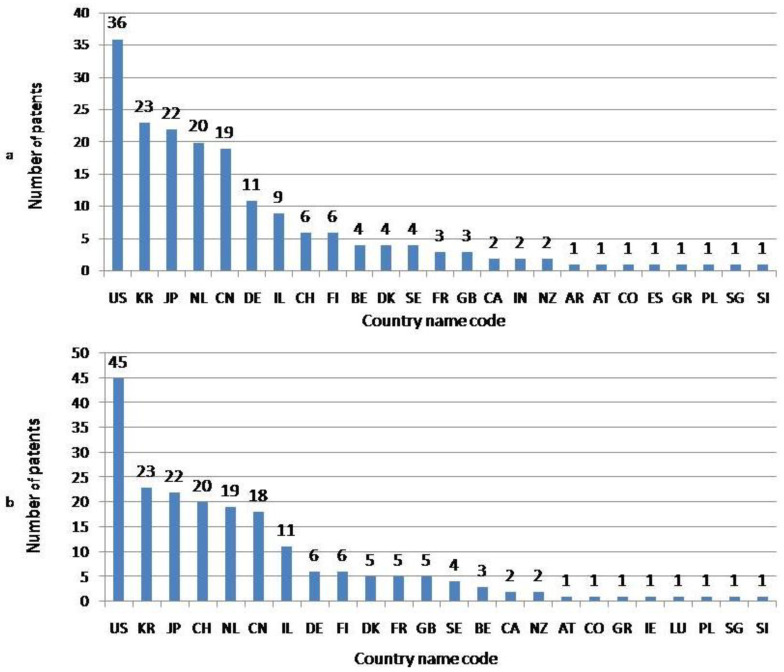
The total number of patents referring to plant-based meat analogues (PBMA) developed in the period 2014–2023 according to first author affiliation (**a**) and affiliation of applicants (**b**). Country name code abbreviations: Argentina (AR), Austria (AT), Belgium (BE), Canada (CA), China (CN), Colombia (CO), Denmark (DK), Finland (FI), France (FR), Germany (DE), Greece (GR), India (IN), Ireland (IR), Israel (IL), Japan (JP), The Netherlands (NL), New Zealand (NZ), Poland (PL), The Republic of Korea (KR), Singapore (SG), Slovenia (SI), Spain (ES), Sweden (SE), Switzerland (CH), Thailand (TH), the United Kingdom of Great Britain and Northern Ireland (GB) and the United States (US).

Altogether, the authors and applicants of patents were affiliated with 27 countries. Among them, the greatest number were affiliated with the United States, the Republic of Korea and Japan. The lowest number of both authors and applicants was in Austria, Colombia, Greece, Poland, Singapore and Slovenia (Figure 3). The H Kruskal–Wallis test confirmed the statistical significance of differences in the spatial distribution of authors (H = 66.92, *p* < 0.001) and applicants for patents (H = 71.59, *p* < 0.001); however, the differences among particular affiliation countries were not significant (Table A2). Considering the applicants, it should be pointed out that solely in the case of four patents were the applications filed by the invention’s authors; in the case of the remaining patents, varied institutions acted as patent applicants. Moreover, regarding 11 patents, the number of applicant institutions exceeded one. In general, among the applicants, 138 institutions were noted (Table 2), and Société des Produits Nestlé S.A. emerged as the leader, followed by Cargill Incorporated; Dsm Ip Assets B.V, and Unilever Ip Holdings B.V.

The International Patent Classification (IPC) codes were given to 177 patents (Table 3). According to Anonymous [38], most IPC codes were classified in Section A (Human Necessities). The most numerous subgroup was A23J (Protein compositions for foodstuffs; working-up proteins for foodstuffs; phosphatide compositions for foodstuffs). Among the most frequent were meat-like textured foods (A23J3/227), followed by vegetable proteins (A23J3/14) and vegetable proteins from soybean (A23J3/16). Another subgroup was A23L (Foods, foodstuffs, or non-alcoholic beverages; their preparation or treatment; modification of nutritive qualities, physical treatment; preservation of foods or foodstuffs, in general). Sporadically mentioned in patent documents were A23D (Edible oils or fats), A23P (Shaping or working of foodstuffs) and A23C (Dairy products), as well as A21D (Treatment, e.g., preservation of flour or dough for baking). Only one IPC code was classified in section B (Performing Operations; Transporting).

### 3.2. Plant Taxa Analysis

**Figure 4:**
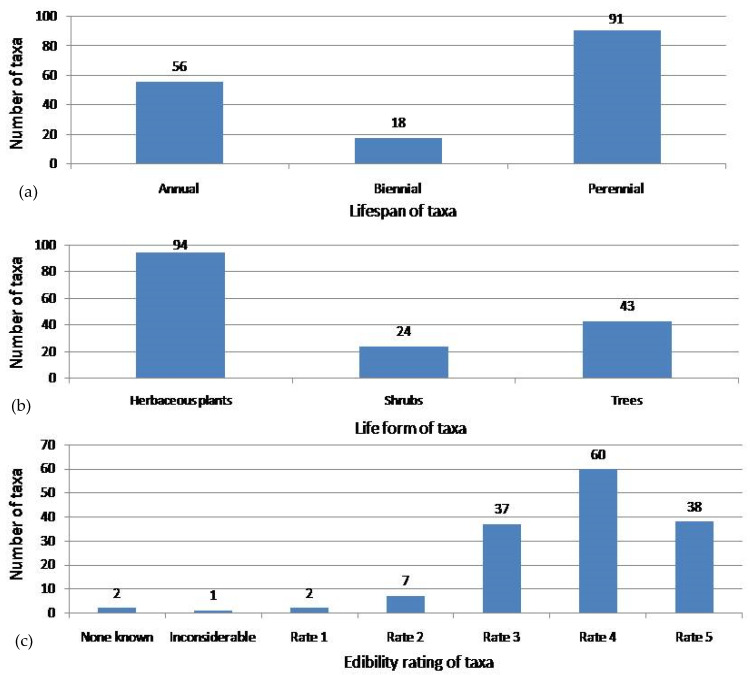
The total number of patents referring to plant-based meat analogues (PBMA) developed in the period 2014–2023 according to the lifespan of taxa (**a**), life form of taxa (**b**) and edibility rating of taxa (**c**) on the basis of The Useful Plants Database [222].

Altogether, taxa from 64 families were recorded in the reviewed patents (Table A3). The greatest number of taxa represented the families Fabaceae (29), Poaceae (16) and Brassicaceae (13). In total, inventors mentioned 184 taxa, including 28 genera, 1 section, 144 species, 5 subspecies and 6 varieties of vascular plants. According to The Useful Plants Database [222], the lifespan, life form, and edibility ratings were assigned to 150 taxa, including species, subspecies, and varieties. Additionally, it should be mentioned that some taxa might be classified into more than one category of lifespan or/and life form. The most abundantly represented were perennials, followed by annuals and biennials. Considering life forms, the majority of taxa mentioned in patents belong to herbaceous plants, while trees and shrubs are less represented. Regarding the edibility rating, it should be pointed out that the most abundant were taxa achieving rate 4, followed by taxa reaching rates 5 and 3. Taxa achieving rates 2 and 1 were rather sporadically mentioned, similar to taxa with inconsiderable or no known edibility value (Figure 4).

The analysis of patents showed that plant taxa are promising sources of proteins, lipids, fibre, polyphenols, starch and gum in meat analogues (Table A4). Altogether, 95 taxa were mentioned as suitable sources of protein (Table 4). Among them, soybean (*Glycine max* L. Merr.), pea (*Lathyrus oleraceus* Lam.), and wheat (*Triticum aestivum* L.) were recorded most frequently. Moreover, numerous inventors indicated that chickpea (*Cicer arietinum* L.), lentil (*Vicia lens* (L.) Coss. & Germ.), rice (*Oryza sativa* L.), potato (*Solanum tuberosum* L.), lupine (*Lupinus* sp. L.), rapeseed (*Brassica napus* L.), oats (*Avena sativa* L.), fava bean (*Vicia faba* L.) and corn (*Zea mays* L.) are also suitable sources of protein. At the same time, 30 taxa such as cherimoya *(Annona cherimola* Mill.), black-eyed bean (*Vigna unguiculata subsp. unguiculata* (L.) Walp.) and others were recorded only once. Among the reviewed patents, 82 taxa were listed as sources of lipids, with rapeseed (*Brassica napus* L.), sunflower (*Helianthus annuus* L.), and soybean (*Glycine max* L. Merr.) belonging to the most frequently mentioned. Also, coconut (*Cocos nucifera* L.), corn (*Zea mays* L.), olive (*Olea europaea* L.), peanut (*Arachis hypogaea* L.), cotton (*Gossypium* sp. L.) and safflower (*Carthamus tinctorium* L.) were recorded by numerous inventors. At the same time, 40 taxa such as fonio (*Digitaria exilis* (Kippist) Stapf), horseradish tree (*Moringa oleifera* Lam.), Ethiopian rapeseed (*Brassica carinata* A.Braun) and others were noted only once (Table 5). In total, 58 taxa acted as a source of fibre in surveyed inventions, with pea (*Lathyrus oleraceus* Lam.), potato (*Solanum tuberosum* L.), as well as psyllium (*Plantago ovata* Forsk) being mentioned most frequently. At the same time, 33 taxa, such as fenugreek *(Trigonellafoenum*-*graecum* L.) and others, were recorded only once (Table 6). Furthermore, 32 taxa were listed by inventors as a source of polyphenols (Table 7). Among them, beet (*Beta vulgaris* L.) and carrot (*Daucus carota* subsp. *sativus* (Hoffm.) Schübl. & G. Martens) were recorded in the greatest number of patents. Simultaneously, 14 taxa, such as amaranth *Amaranthus* sp. L. and gooseberry *Ribes uva-crispa* L. were noticed only once. Altogether, 32 taxa were recorded as a source of starch in meat substitutes, with corn (*Zea mays* L.), potato (*Solanum tuberosum* L.), wheat (*Triticum aestivum* L.), and tapioca (*Manihot esculenta* Crantz.) most frequently noted (Table 8). Also, rice (*Oryza sativa* L.), pea (*Lathyrus oleraceus* Lam.), and sweet potato (*Ipomoea batatas* (L.) Lam.) were mentioned in several inventions. At the same time, 11 taxa, such as pigeon pea *Cajanus cajan* (L.) Millsp and quinoa *Chenopodium quinoa* Willd. were recorded only one time. In total, 10 taxa were specified as gum sources (Table 9). Among them, locust bean (*Ceratonia siliqua* L.), guar (*Cyamopsistetragonoloba* (L.) Taub), konjac (*Amorphophallus konjac* K. Koch) and tragacanth (*Astragalus gummifer* Labill.) were the most frequently recorded, while cassia (*Cassia* sp. L.) and axlewood *(Terminalia anogeissiana* Gere & Boatwr.) were noted only once.

## 4. Discussion

The increasing number of patents in the years 2020–2022 might respond to the globally observed tendency of growing consumer demand for plant-based meat analogues. Numerous authors have argued that in recent decades, the European [223], American [224], African [225] and Australian [226] markets for plant-based meat substitutes have been experiencing unprecedented growth. The lower number of patents recorded in the year 2023, noticed in the present study, corresponds with other surveys of patents showing the diminishing number of published inventions in the last years of investigation periods [227,228,229]. Such a phenomenon might be related to the time involved in waiting for indexation in the databases, reaching 18 months in the case of the Espacenet [230] database and The United States Patent and Trademark Office [231].

The performed study’s evidence that the greatest number of inventions was developed by research teams consisting of two authors corresponds with the worldwide tendency of a transition of scientific research patterns in the natural sciences from individual research to teamwork [232]. On the other hand, the observed gradual decline in patent numbers with a growing number of authors above three is intriguing, similar to the statistically confirmed remarkably lower number of patents invented by research teams consisting of 8 or more authors. Such a phenomenon might be explained by the findings of Azoulay [233] and Osório and Bornmann [234], who argued that research conducted by small teams is more likely to lead to significant results than research by large teams. According to the aforementioned authors, researchers in possession of potentially breakthrough research ideas prefer to keep teams as small as possible.

The lack of forward citations of the majority of surveyed patents might be worrying, especially considering the findings of Svensson [235], who argued that forward citations used as a measure of patent value indicate the existence of downstream research efforts, a potential market for a patent and commercialization of an invention. Additionally, Fischer and Leidinger [236] show that forward citations are positively related to patent value (measured as patent auction prices). The lack of citations observed in the investigation might be explained by the time needed to receive them, which seems to be insufficient in the case of recently issued patents. This thesis is consistent with the statistically evidenced significant negative correlation between the year of patent publication and the number of forward citations.

The investigation showed that the majority of authors and applicants are affiliated with the United States, which corresponds to the fact that this country is the leader in the production of plant-based meat analogues [237]. At the same time, it is worth mentioning that authors affiliated with the United States developed the greatest number of patented food products making use of kidney bean [228], maize [238] and soybean [239] and developed the majority of all patents filed for alternative proteins [240]. Considering this, it seems surprising that among the applicants for the patented plant-based meat analogues surveyed, one of the most important companies in the US food industry, Beyond Meat [12], was not noted, while the company Impossible Foods [241] acted as a patent applicant only twice. On the other hand, other US companies common in the market of plant-based meat analogues, such as Cargill Inc. and Unilever Ip Holdings B.V., acted as applicants in several patent documents. Simultaneously, it is worth mentioning that the observed leading position among patent applicants of the Swiss company Société des Produits Nestlé S.A. confirms other market studies reviewed by Boukid [9].

The most frequently noticed International Classification codes, *Meat-like textured foods* (A23J3/227), followed by *Vegetable proteins* (A23J3/14) and *Vegetable proteins from soybean* (A23J3/16), seem to reflect the use of vegetable proteins from soybean, often mentioned in surveyed patent descriptions. Considering the results of other patent reviews referring to the application of plant proteins in various food products (e.g., [227,229,240,242] and literature cited there), the use of soybean and the other herbaceous plants, mainly annual taxa from Fabaceae (such as pea, chickpea, lentil, lupine, fava bean) and Poaceae (e.g., wheat, rice, oats, corn) families as a source of protein in plant-based meat analogues is not surprising. However, although most plant protein sources provide the required amounts of essential amino acids for human needs, plant proteins are often recognized as incomplete or nutritionally inferior to animal proteins [243]. As stated by the aforementioned authors, depending on the source, plant proteins may be deficient in some essential amino acids, e.g., cereals usually contain low levels of lysine, while legumes have a deficiency in sulfur amino acids. Moreover, there are many other reasons why plant proteins are still insufficiently applied as human food, such as difficulties in maximising their physical functionality due to their large molecular weight and size and poor solubility in water, as well as the economic cost associated with isolation and recovery of protein fractions [244]. Furthermore, the performed review of patents shows that apart from proteins, numerous annuals and some perennials are mentioned as a main source of lipids (rapeseed, sunflower, soybean), fibre (pea, potato, psyllium), polyphenols (beet, carrot), starch (corn, potato, wheat, Manihot, rice, pea, sweet potato), as well as gum (locust bean, guar, konjac, tragacanth) is consistent with findings reporting the considerable value of the aforementioned species as sources of necessary constituents in the human diet [31,245,246,247,248,249,250,251,252,253,254,255,256,257,258,259]. At the same time, it is worth mentioning that the use of some plant ingredients, such as coconut oil, might be controversial [11]. Apart from nutritive value, the aforementioned constituents play other important roles in meat analogues. Egbert and Borders [260] pointed out that vegetable lipids act as binding agents and provide lubrication to the modern meat analogue. The addition of oil or fat gives juiciness, tenderness, and particular flavour in a meat analogue, which is a unique attribute of a food recipe. In addition, starches are commonly used as fillers to improve the texture and consistency of PBMAs, while fibres and gums can act as binding agents to enhance product stability, thickness, and consistency and reduce cooking loss [9,261,262].

On the other hand, the slight use of many annual and perennial species (herbaceous plants, shrubs and trees) in plant-based meat analogues, despite their substantial content of valuable constituents [263], as well as a high edibility rating, might be linked to their underutilization in many regions of the world. Such a phenomenon has already been confirmed in the case of amaranth, bambara nut, black-eyed bean, cherimoya, Ethiopian rapeseed, fonio, gooseberry, horseradish tree, kenaf, pigeon pea and taro, among others [264,265,266,267,268,269], the wide variety of nutrient-rich plant species (including the aforementioned taxa) used in earlier times is nowadays neglected for reasons including problems with production and harvesting, biotic factors (e.g., insects, diseases), abiotic issues (e.g., temperature, soil fertility, waterlogging, drought), poor economic attractiveness, the lack of policy recommendations and many others.

## 5. Conclusions

The observed increasing number of patents in the years 2020–2022 might respond to the worldwide observed tendency of growing consumer demand for plant-based meat analogues. The greatest number of inventions developed by research teams consisting of two authors correspond with the worldwide tendency of the transition of scientific research patterns in the natural sciences from individual research to teamwork. However, the gradual decline of patent numbers with the number of inventors growing above three might be linked to the fact evidenced in the literature that scientists in possession of potentially breakthrough research ideas prefer to work in small teams. The lack of forward citations of the majority of patents might be a cause for worry; however, the evidenced negative correlation between the patent publication year and the number of citations gives hope of receiving citations, particularly for recently issued patents. The majority of authors and applicants affiliated with the United States agree that this country is the chief producer of plant-based meat analogues. Despite the fact that important US food industry companies acted as patent applicants, The Société des Produits Nestlé S.A., affiliated with Switzerland, emerged as the leader. The majority of taxa mentioned in plant-based meat analogues presented a high edibility rating and belonged to perennials, mainly herbaceous plants. However, the most frequently mentioned source of protein was annual soybean, as well as other short-lived taxa from the Fabaceae (pea, chickpea, lentil, lupine, fava bean) and Poaceae (e.g., wheat, rice, oats, corn) families, whilst numerous annual and perennial species (herbaceous plants, shrubs and trees) were frequently noticed as sources of lipids, polyphenols, starch, fibre and gum. At the same time, the slight use of numerous taxa, despite their substantial content of valuable constituents, as well as considerable edibility rating, presumably might be linked with their underutilization. Considering the great potential of useful plant species shown in the presented review, it might be stated that further investigations seem to be strongly desirable. Their main direction should be focused on searching for novel plant-based meat analogues based on underutilized, promising plant sources and investigating their effects on people’s performance, especially with increased physical activity.

## Figures and Tables

**Table 1:** Number of forward citations of patents referring to plant-based meat analogues (PBMA) developed in the period 2014–2023.

Number of Forward Patent Citations	Number of Patents with Particular Number of Forward Citations
0	73
1	30
2	19
3	7
4	6
5	2
6	10
7	5
8	4
9	1
10	2
11	5
12	2
13	3
15	1
16	1
17	1
19	1
23	3
30	1
46	1
49	1
57	1
100	1

**Table 2:** The ranking of leading patent applicants with the number of patent applications.

Applicant Institution	Number of Patent Applications
Société des Produits Nestlé S.A.	17
Cargill, Incorporated	7
Dsm Ip Assets B.V.	6
Unilever Ip Holdings B.V.	6
Fujifilm Corp	5
Redefine Meat Ltd.	5
AAK AB	4
Fuji Oil Co., Ltd.	4
Northeast Agricultural University	4
Conopco, Inc.	3
Gold&Green Foods Oy	3
Kongju University Industry-Academic Cooperation Foundation	3
Konkuk University Industry-Academic Cooperation Foundation	3
Motif Foodworks, Inc.	3

**Table 3:** The International Patent Classification (IPC) codes mentioned in patent applications refer to plant-based meat analogues (PBMA) developed in the period 2014–2023.

Classification Code	Description	Number of Patents
A21D2/36	Vegetable material	2
A23C20/025	Cheese substitutes containing neither milk components nor caseinate nor lactose, as sources of fats, proteins or carbohydrates mainly containing proteins from pulses or seeds	1
A23D7/0053	Compositions other than spreads	6
A23D9/04	Working-up	1
A23J1/006	Obtaining protein compositions for foodstuffs; Bulk opening of eggs and separation of yolks from whites from vegetable materials	2
A23J1/14	Obtaining protein compositions for foodstuffs; Bulk opening of eggs and separation of yolks from whites from leguminous or other vegetable seeds; from press-cake or oil-bearing seeds	1
A23J3/00	Working-up of proteins for foodstuffs	3
A23J3/14	Vegetable proteins	23
A23J3/16	Vegetable proteins from soybean	12
A23J3/18	Vegetable proteins from wheat	3
A23J3/20	Proteins from microorganisms or unicellular alga	1
A23J3/22	Working-up of proteins for foodstuffs by texturising	2
A23J3/225	Texturised simulated foods with high protein content	5
A23J3/227	Meat-like textured foods	3
A23J3/26	Working-up of proteins for foodstuffs by texturising using extrusion or expansion	10
A23J3/346	Working-up of proteins for foodstuffs by hydrolysis using chemical agents using enzymes of vegetable protein	1
A23L11/00	Pulses, i.e., fruits of leguminous plants, for the production of food; Products from legumes; Preparation or treatment thereof	1
A23L11/05	Mashed or comminuted pulses or legumes; Products made therefrom	1
A23L11/07	Soya beans, e.g., oil-extracted soya bean flak es	1
A23L11/34	Removing undesirable substances, e.g., bitter substances, using chemical treatment, adsorption or absorption	1
A23L13/10	Meat meal or powder; granules, agglomerates or flakes	1
A23L13/40	Meat products; Meat meal; Preparation or treatment thereof containing additives	1
A23L13/426	Addition of proteins, carbohydrates or fibrous material from vegetable origin other than sugars or sugar alcohols	2
A23L13/60	Comminuted or emulsified meat products, e.g., sausages; Reformed meat from comminuted meat product	1
A23L13/67	Reformed meat products other than sausages	1
A23L13/72	Tenderised or flavoured meat pieces; Macerating or marinating solutions specially adapted, therefore using additives, e.g., by injection of solutions	1
A23L19/00	Products from fruits or vegetables; preparation or treatment thereof	1
A23L19/01	Instant products; powders; flakes; granules	1
A23L19/12	Products from fruits or vegetables; preparation or treatment thereof of tuberous or like starch-containing root crops of potatoes	1
A23L27/215	Synthetic spices, flavouring agents or condiments containing amino acids heated in the presence of reducing sugars, e.g., Maillard’s non-enzymatic	1
A23L27/26	Meat flavours	1
A23L29/015	Inorganic compounds	1
A23L29/04	Fatty acids or derivatives	1
A23L29/10	Foods or foodstuffs containing additives; preparation or treatment thereof containing emulsifiers	1
A23L29/20	Foods or foodstuffs containing additives; preparation or treatment thereof containing gelling or thickening agents	1
A23L29/212	Starch; modified starch; starch derivatives, e.g., esters or ethers	1
A23L29/244	Foods or foodstuffs containing additives; preparation or treatment thereof containing gelling or thickening agents of vegetable origin from corms, tubers or roots, e.g., glucomannan	1
A23L29/256	Foods or foodstuffs containing additives; preparation or treatment thereof containing gelling or thickening agents of vegetable origin from seaweeds, e.g., alginates, agar or carrageenan	2
A23L29/262	Cellulose; derivatives thereof, e.g., ethers	3
A23L29/269	Foods or foodstuffs containing additives; Preparation or treatment thereof containing gelling or thickening agents of microbial origin, e.g., xanthan or dextran	1
A23L33/10	Modifying nutritive qualities of foods; dietetic products; preparation or treatment thereof using additives	1
A23L33/105	Plant extracts, their artificial duplicates or their derivatives	1
A23L33/115	Fatty acids or derivatives thereof; fats or oils	1
A23L33/16	Inorganic salts, minerals or trace elements	2
A23L33/18	Peptides; protein hydrolysates	1
A23L33/185	Vegetable proteins	15
A23L35/10	Emulsified foodstuffs	1
A23L5/43	Addition of dyes or pigments, e.g., in combination with optical brighteners using naturally occurring organic dyes or pigments, their artificial duplicates or their derivatives	3
A23L5/44	Addition of dyes or pigments, e.g., in combination with optical brighteners using naturally occurring organic dyes or pigments, their artificial duplicates or xanthophylls	1
A23L7/198	Dry unshaped, finely divided cereal products, not provided for in groups A23L7/117–A23L7/196 and A23L29/00, e.g., meal, flour, powder, dried cereal	1
A23P10/25	Agglomeration or granulation by extrusion or by pressing, e.g., through small holes, through sieves or between surfaces	1
A23P20/10	Coating with edible coatings, e.g., with oils or fats	1
A23P20/20	Making of laminated, multi-layered, stuffed or hollow foodstuffs, e.g., by wrapping in preformed edible dough sheets or in edible food containers	1
A23P30/20	Extruding	2
B29C48/022	Extrusion moulding, i.e., expressing the moulding material through a die or nozzle which imparts the desired form; apparatus characterised by the choice of material	1

**Table 4:** Plant taxa are mentioned as a source of protein in patent documents referring to plant-based meat analogues (PBMA) developed in the period 2014–2023. Common names of plant taxa are presented in Table A3.

Plant Taxa	Number of Patents
*Glycine max* L. Merr.	139
*Lathyrus oleraceus* Lam.	118
*Triticum aestivum* L.	89
*Cicer arietinum* L.	60
*Vicia lens* (L.) Coss. & Germ.	55
*Oryza sativa* L.	46
*Solanum tuberosum* L.	44
*Lupinus* sp. L.	43
*Brassica napus* L.	42
*Avena sativa* L.	40
*Vicia faba* L., *Zea mays* L.	38
*Helianthus annuus* L.	34
*Phaseolus vulgaris* L	34
*Vigna radiata* (L.) R. Wilczek	31
*Arachis hypogaea* L.	29
*Hordeum vulgare* L.	25
*Cannabis sativa* L., *Chenopodium quinoa* Willd.	20
*Sesamum indicum* L.	18
*Gossypium* sp. L.	14
*Medicago sativa* L.	13
*Salvia hispanica* L.	12
*Prunus amygdalus* Batsch, *Secale cereale* L.	11
*Fagopyrum esculentum* Moench.	10
*Cucurbita pepo* L., *Vigna unguiculata* subsp. *stenophylla* (Harv.) Maréchal, Mascherpa and Stainier	9
*Camelina sativa* (L.) Crantz	8
*Cenchrus americanus* (L.) Morrone	7
*Amaranthus* sp. L., *Brassica oleracea* var. *italica* Plenck, *Cocos nucifera* L., *Ipomoea batatas* (L.) Lam., *Manihot esculenta* Crantz., *Sorghum bicolor* L. Moench, *Triticum spelta* L.	6
*Anacardium occidentale* L., *Corylus avellana* L., *Trifolium* sp. L.	5
*Brassica oleracea* var. *botrytis* L., *Cynaracardunculus* var. *scolymus* L., *Musa* sp., *Vignaangularis* (Willd.) Ohwi and H. Ohashi.	4
*Artocarpus heterophyllus* Lam., *Asparagus officinalis* L., *Brassica oleracea* L., *Ceratonia siliqua* L., *Juglans regia* L., *Macadamia* sp. F. Muell., *Oleaeuropaea* L., *Perseaamericana* Mill., *Phaseoluslunatus* L., *Pistacia vera* L., *Prosopis* sp. L.	3
*Bertholletia excelsa* Humb. & Bonpl., *Cajanus cajan* (L.) Millsp, *Digitaria exilis* (Kippist) Stapf, *Eragrostis tef* (Zucc.) Trotter, *Oxalis tuberosa* Molina, *Oxycoccus* sp. Hill., *Spinacia oleracea* L., *Theobroma cacao* L., *Zizania* sp. L.	2
*Actinidia* sp. Lindl., *Annona cherimola* Mill., *Apium graveolens* L., *Artocarpus camansi* Blanco, *Attalea speciosa* Mart., *Beta vulgaris* L. subsp. *vulgaris*, *Brassica juncea* (L.) Czern., *Brassica oleracea* var. *gongylodes* L., *Carya illinoinensis* (Wangenh.) K. Koch, *Celosia* sp. L., *Chenopodium pallidicaule* Aellen, *Colocasia esculenta* (L.) Schott, *Cyamopsis tetragonoloba* (L.) Taub, *Daucus carota* subsp. *sativus* (Hoffm.) Schübl. & G. Martens, *Lablab purpureus* (L.) Sweet, *Linum usitatissimum* L., *Maranta arundinacea* L., *Morus* sp. L., *Nicotiana* sp. L., *Pinus* sp. L., *Plukenetiavolubilis* L., *Psidium guajava* L., *Rhamphospermum nigrum* L. Al-Shehbaz, *Ribes uva-crispa* L., *Sinapis alba* L., *Triticum dicoccon* (Schrank) Schübl., *Triticum turgidum* subsp. *turanicum* (Jakubz.) Á.Löve, *Vicia* sp., *Vignasubterranea* (L.) Verdc., *Vigna unguiculata* subsp. *unguiculata* (L.) Walp., *Tamarindus indica* L.	1

**Table 5:** Plant taxa are mentioned as a source of lipids in patent documents referring to plant-based meat analogues (PBMA) developed in the period 2014–2023. Common names of plant taxa are presented in Table A3.

Plant Taxa	Number of Patents
*Brassica napus* L.	76
*Helianthus annuus* L.	72
*Glycine max* L. Merr.	70
*Cocos nucifera* L.	60
*Olea europaea* L., *Zea mays* L.	59
*Arachis hypogaea* L.	43
*Gossypium* sp. L.	41
*Carthamus tinctorium* L.	39
*Sesamum indicum* L.	28
*Oryza sativa* L.	27
*Vitellaria paradoxa* C.F.Gaertn.	22
*Camelina sativa* (L.) Crantz	20
*Prunus amygdalus* Batsch, *Theobroma cacao* L.	19
*Juglans regia* L.	17
*Persea americana* Mill., *Vitis vinifera* L.	14
*Triticum aestivum* L.	10
*Attalea speciosa* Mart., *Corylus avellana* L., *Macadamia* sp. F.Muell.	9
*Mangifera indica* L.	9
*Linum usitatissimum* L., *Ribes nigrum* L.	8
*Borago officinalis* L.	6
*Carya illinoinensis* (Wangenh.) K.Koch, *Cucurbita pepo* L., *Hippophae rhamnoides* L., *Perilla frutescens* (L.) Britton, *Anacardium occidentale* L., *Cannabis sativa* L., *Oenothera biennis* L., *Shorea stenoptera* Burck.	5
*Avena sativa* L., *Camellia sinensis* (L.) Kuntze, *Ceiba pentandra* (L.) Gaertn., *Coriandrum sativum* L., *Euterpe oleracea* Mart., *Papaver* sp. L., *Pistacia vera* L., *Ricinus communis* L.	3
*Allanblackia floribunda* Oliv., *Amaranthus* sp. L., *Caryocar brasiliense* Cambess., *Ceratonia siliqua* L., *Chenopodium quinoa* Willd., *Cucurbita foetidissima* Kunth, *Garcinia indica* Choisy, *Guizotia abyssinica* (L. f.) Cass, *Lallemantiaroyleana* Benth. in Wall., *Limnanthes* sp. R.Br., *Pinus* sp. L., *Prunus armeniaca* L., *Sideroxylon spinosum* L., *Simmondsia chinensis* (Link) C.K. Schneid.	2
*Abelmoschus esculentus* (L.) Moench, *Actinidia* sp. Lindl., *Allium cepa* L., *Bertholletia excelsa* Humb. & Bonpl., *Brassica carinata* A. Braun, *Brassica rapa* (L.) Koch., *Calendula officinalis* L., *Citrullus lanatus* (Thunb.) Matsum. & Nakai, *Citrus × tangerina* Tanaka, *Coffea canephora* Pierre ex A. Froehner, *Cynara cardunculus* var. *scolymus* L., *Daucus carota* subsp. *sativus* (Hoffm.) Schübl. & G. Martens, *Digitaria exilis* (Kippist) Stapf, *Fagus* sp. L., *Hibiscus cannabinus* L., *Hordeum vulgare* L., *Hydnophytum papuanum* Becc., *Jatropha* sp. L., *Lupinus* sp. L., *Medicago sativa* L., *Moringa oleifera* Lam., *Prunus domestica* L., *Santalum yasi* Bertero, *Shorea robusta* Roth, *Sorghum bicolor* L. Moench, *Vernicia fordii* (Hemsl.) Airy Shaw.	1

**Table 6:** Plant taxa mentioned as a source of fibre in patent documents referring to plant-based meat analogues (PBMA) developed in the period 2014–2023. Common names of plant taxa are presented in Table A3.

Plant Taxa	Number of Patents
*Lathyrus oleraceus* Lam.	12
*Solanum tuberosum* L.	10
*Plantago ovata* Forsk	9
*Malus domestica* Borkh.	8
*Avena sativa* L.	7
*Daucus carota* subsp. *sativus* (Hoffm.) Schübl. & G. Martens, *Triticum aestivum* L.	6
*Ipomoea batatas* (L.) Lam., *Zea mays* L.	5
*Lupinus* sp. L.	4
*Manihot esculenta* Crantz., *Musa* sp., *Oryza sativa* L., *Vicia faba* L.	3
*Beta vulgaris* L., *Beta vulgaris* L. subsp. *vulgaris, Cicer arietinum* L., *Citrus* sp. L., *Cucurbita pepo* L., *Hordeum vulgare* L., *Saccharum* sp. L., *Vigna radiata* (L.) R. Wilczek	2
*Acer* sp. L., *Agave sisalana* Perrine, *Amorphophallus konjac* K. Koch, *Ananas comosus* (L.) Merr., *Apium graveolens* L., *Artocarpus heterophyllus* Lam., *Brassica oleracea* L., *Brassica oleracea* var. *italica* Plenck, *Cucurbita pepo* var. *cylindrica* L., *Cajanus cajan* (L.) Millsp, *Cannabis sativa* L., *Cocos nucifera* L., *Codonopsis lanceolata* (Siebold & Zucc.) Trautv., *Colocasia esculenta* (L.) Schott, *Fagopyrum esculentum* Moench, *Glycine max* L. Merr., *Gossypium* sp. L., *Helianthus annuus* L., *Linum usitatissimum* L., *Malva* sp. L., *Medicago sativa* L., *Musatextilis* Née, *Oxalis tuberosa* Molina, *Petroselinum crispim* (Mill.) Fuss, *Phaseolus vulgaris* L., *Raphanus sativus* L., *Secalecereale* L., *Solanumlycopersicum* L., *Sorghumbicolor* L. Moench, *Spinaciaoleracea* L., *Trigonellafoenum*-*graecum* L., *Tussilago farfara* L., *Vicia lens* (L.) Coss. & Germ.	1

**Table 7:** Plant taxa mentioned as a source of polyphenols in patent documents referring to plant-based meat analogues (PBMA) developed in the period 2014–2023. Common names of plant taxa are presented in Table A3.

Plant Taxa	Number of Patents
*Beta vulgaris* L.	11
*Daucus carota* subsp. *sativus* (Hoffm.) Schübl. & G. Martens	7
*Raphanus sativus* L.	5
*Solanum lycopersicum* L.	4
*Brassica oleracea* L., *Camellia sinensis* (L.) Kuntze, *Carthamus tinctorium* L., *Fragaria × ananasa* Duchesne, *Punica granatum* L., *Ribes nigrum* L., *Rubus idaeus* L.	3
*Bixa orellana* L., *Capsicum annuum* L., *Gardenia* sp. J.Ellis, *Hibiscus* sp. L., *Ipomoea batatas* (L.) Lam., *Solanum tuberosum* L., *Vaccinium* sect. *cyanococcus* Rydb.	2
*Amaranthus* sp. L., *Artemisia vulgaris* L., *Coffea canephora* Pierre ex A. Froehner, *Cucurbita pepo* L., *Curcuma longa* L., *Malus domestica* Borkh., *Oxycoccus* sp. Hill., *Ribes rubrum* L., *Ribes uva-crispa* L., *Rubus × loganobaccus* L.H. Bailey, *Sambucus* sp. L., *Spinacia oleracea* L., *Theobroma cacao* L., *Vitis vinifera* L.	1

**Table 8:** Plant taxa mentioned as a source of starch in patent documents referring to plant-based meat analogues (PBMA) developed in the period 2014–2023. Common names of plant taxa are presented in Table A3.

Plant Taxa	Number of Patents
*Zea mays* L.	34
*Solanum tuberosum* L.	31
*Triticum aestivum* L.	23
*Manihot esculenta* Crantz.	20
*Oryza sativa* L.	17
*Lathyrus oleraceus* Lam.	14
*Ipomoea batatas* (L.) Lam.	12
*Maranta arundinacea* L.	9
*Hordeum vulgare* L., *Sorghum bicolor* L. Moench	8
*Avena sativa* L.	6
*Oxalis tuberosa* Molina, *Vigna radiata* (L.) R. Wilczek	5
*Colocasia esculenta* (L.) Schott, *Musa textilis* Née	4
*Amorphophallus konjac* K. Koch, *Artocarpus altilis* (Parkinson) Fosberg, *Cicer arietinum* L., *Plantago major* L., *Vicia lens* (L.) Coss. & Germ.	3
*Fagopyrum esculentum* Moench, *Vicia faba* L.	2
*Arracacia xanthorrhiza* Bancr., *Cajanus cajan* (L.) Millsp, *Ceratonia siliqua* L., *Chenopodium quinoa* Willd., *Cyamopsis tetragonoloba* (L.) Taub, *Erythronium japonicum* Decne., *Nelumbo nucifera* Gaertn., *Phaseolus vulgaris* L., *Pueraria montana* (Lour.) Merr., *Secale cereale* L.	1

**Table 9:** Plant taxa mentioned as a source of gum in patent documents referring to plant-based meat analogues (PBMA) developed in the period 2014–2023. Common names of plant taxa are presented in Table A3.

Plant Taxa	Number of Patents
*Cyamopsis tetragonoloba* (L.) Taub	24
*Ceratonia siliqua* L.	23
*Amorphophallus konjac* K. Koch	14
*Astragalus gummifer* Labill.	10
*Tara* sp. Molina	5
*Sterculia urens* Roxb.	4
*Acacia* sp. Mill., *Trigonella foenum-graecum* L.	3
*Cassia* sp. L., *Terminalia anogeissiana* Gere & Boatwr.	1

## Data Availability

Data are presented in the paper.

## Appendix A

**Table A1:** The characteristics of reviewed patented plant-based meat analogues (PBMA) developed in the years 2014–2023.

	First Inventor	Number of Inventors	First Author Affiliation	Year	Patent Title	International Patent Classification	Applicant Name	Applicant Affiliation	Number of Patent Citations	Reference
1	Janvary, L.	2	DE	2014	Meat texturizer	A23J3/227 Meat-like textured foods	Suedzucker AG	DE	6	[39]
2	Liu, X.	2	CN	2014	Meat substitute stuffing dessert and preparation method thereof	.	Jinggangshan Jingxiang Mushroom Science & Technology Co., Ltd.	CN	7	[40]
3	Ma, Y.	1	CN	2014	Meat substitute and production method thereof	A23L11/05 Mashed or comminuted pulses or legumes; Products made therefrom	Xiangcheng Linghua Flour Co., Ltd.	CN	15	[41]
4	Nakano, Y.	1	JP	2014	Textured vegetable protein material and substitute for thin meat slices using same	A23J3/14 Vegetable proteins	Fuji Oil Co., Ltd.	JP	6	[42]
5	Redl, A.	1	BE	2014	A proteinaceous meat analogue having an improved texture and an extended shelf-life	A23J3/18 Vegetable proteins from wheat	Syral Belgium Nv	BE	19	[43]
6	Cully, K.J.	3	US	2015	Non-meat food products having appearance and texture of cooked meat	A21D2/36 Vegetable material	Nestec S.A.	CH	16	[44]
7	Eisner, P.	5	DE	2015	Fiber preparation from lupine seeds	A21D2/36 Vegetable material	Fraunhofer Gesellschaft zur Forderung der AngewandtenForschung eV	DE	0	[45]
8	Kivelä, R.	2	FI	2015	A method of manufacturing a textured food product and a texturized food product	A23J3/26 Working-up of proteins for foodstuffs by texturising using extrusion or expansion	Gold & Green Foods Oy	FI	23	[46]
9	Kubara, M.	2	PL	2015	Basis for vegetable meat substitute	A23L33/16 Inorganic salts, minerals or trace elements	Kubura, Spółka Jawna	PL	5	[47]
10	Nakano, Y.	1	JP	2015	Shrimp meat substitute and process for producing same	A23J3/16 Vegetable proteins from soybean	Fuji Oil Co., Ltd.	JP	6	[48]
11	Varadan, R.	9	IN	2015	Ground meat replicas	A23L33/185 Vegetable proteins	Impossible Foods Inc.	US	100	[49]
12	Vrljic, M.	8	US	2015	Methods and compositions for consumables	A23L27/26 Meat flavours	Impossible Foods Inc.	US	57	[50]
13	Redl, A.	5	BE	2016	Highly nutritious proteinaceous meat analogue having improved texture and extended shelf-life	A23J3/18 Vegetable proteins from wheat	Syral Belgium Nv	BE	12	[51]
14	Lee, S.	2	NZ	2016	Meat substitute	A23J3/227 Meat-like textured foods	Sunfed Limited	NZ	30	[52]
15	Trottet, G.	5	CH	2016	A process for preparing a meat-analogue food product	A23J3/26 Working-up of proteins for foodstuffs by texturising using extrusion or expansion	Société des Produits Nestlé S.A.	CH	49	[53]
16	Jones, A.	4	US	2017	Ready-to-eat, shelf-stable tater tot-type snack food	A23L19/12 Products from fruits or vegetables; Preparation or treatment thereof of tuberous or like starch containing root crops of potatoes	Continental Mills Inc.	US	0	[54]
17	Mitchell, M.	1	US	2017	System and method for preparing ready-to-eat plant-based foods	A23L19/01 Instant products; Powders; Flakes; Granules	Application filed by author	US	2	[55]
18	Nakai, S.	1	JP	2017	Dried meat-like foods	.	Fuji Oil Co., Ltd.	JP	1	[56]
19	Reifen, R.	3	IL	2017	Chickpea protein concentrate	A23L11/34 Removing undesirable substances, e.g., bitter substances, using chemical treatment, adsorption or absorption	Yissum Research Development Company Of The Hebrew University Of Jerusalem Ltd.	IL	13	[57]
20	Beekmans, L.Ch.H.	3	NL	2018	Shaped vegetarian meat product	A23J3/227 Meat-like textured foods	Unilever IP Holdings BV	US	10	[58]
21	Christensen, T.	3	DK	2018	A method for production of vegetable meat substitute with improved texture	A23J3/227 Meat-like textured foods	Dragsbaek A.S.	DK	23	[59]
22	Frank, J.L.	2	GB	2018	A foodstuff	A23L29/262 Cellulose; Derivatives thereof, e.g., ethers	Meatless Farm Ltd.	GB	3	[60]
23	Hong, G-P.	3	KR	2018	A meat analogue having the imitated fascia and a process for the preparation thereof	A23J3/227 Meat-like textured foods	Sejong University Industry-Academic Cooperation Foundation	KR	2	[61]
24	Kaukonen, O.	3	FI	2018	Meat substitute and production thereof from plant material	A23J3/14 Vegetable proteins	Raisio Nutrition Ltd.	FI	13	[62]
25	Maldonado, O.	3	US	2018	Dairy-based meat substitute and methods of producing the same	A23J3/227 Meat-like textured foods	Land OLakes Inc.	US	1	[63]
26	Mellema, M.	2	NL	2018	Meat alternative comprising aqueous gelling composition	A23J3/227 Meat-like textured foods	Unilever N.V./Unilever Plc/Conopco, Inc./D/B/A Unilever	NL/GB/US/US	8	[64]
27	Ogawa, J.	1	JP	2018	Livestock meat-like processed food, its production method and livestock meat-like processed food additive	A23J3/227 Meat-like textured foods	Japan Maize Products Co., Ltd./Nihon Shokuhin Kako Co., Ltd.	JP/JP	6	[65]
28	Park, T-J.	1	KR	2018	Meal substitute for cereals as main ingredients, manufacturing method thereof	A23L7/198 Dry unshaped finely divided cereal products, not provided for in groups A23L7/117-A23L7/196 and A23L29/00, e.g., meal, flour, powder, dried cereal creams or extracts	Erestory Co., Ltd.	KR	0	[66]
29	Ryu, Ki-H.	2	KR	2018	Texturized Vegetable Protein extruded with green tea	A23J3/227 Meat-like textured foods	Kongju University Industry-Academic Cooperation Foundation	KR	2	[67]
30	Choi, M-J.	4	KR	2019	Preparation method for meat analogue comprising emulsion as a substitute for meat fat	A23J3/16 Vegetable proteins from soybean	Konkuk University Industry-Academic Cooperation Foundation	KR	6	[68]
31	Fernandez Fares, I.	3	CH	2019	A process for making a plant-based product	A23J3/227 Meat-like textured foods	Société des Produits Nestlé S.A.	CH	23	[69]
32	Jiang, Z.	4	FI	2019	A meat replacement product, a method and a twin-screw extruder for manufacturing the same	B29C48/022 Extrusion moulding, i.e., expressing the moulding material through a die or nozzle which imparts the desired form; Apparatus therefore characterised by the choice of material	Gold & Green Foods Oy	FI	8	[70]
33	Nakano, T.	2	JP	2019	Method for producing raw fish meat alternative material	.	Fuji Oil Co Ltd., Fuji Oil Holdings Inc.	JP	2	[71]
34	Tulbek, M.	4	CA	2019	Pulse-based bread crumb, coating and pre-dust analog process for manufacturing the same	A23P20/10 Coating with edible coatings, e.g., with oils or fats	Agt Food And Ingredients Inc.	CA	3	[72]
35	Wang, X.	6	CN	2019	One kind holding odor type meaty food and its processing method	A23C20/025 Cheese substitutes containing neither milk components, nor caseinate, nor lactose, as sources of fats, proteins or carbohydrates mainly containing proteins from pulses or oilseeds	Northeast Agricultural University	CN	4	[73]
36	Weaver, Ch.C.	2	GR	2019	Foodstuff with meat substitute	A23J3/14 Vegetable proteins	A. And X. Yfantis Ave	GR	0	[74]
37	Verkuijl, B.J.V.	2	NL	2019	Meat analogue product and method	A23J3/26 Working-up of proteins for foodstuffs by texturising using extrusion or expansion	Bunge Loders Croklaan B.V.	NL	7	[75]
38	Ben-Shitrit, E.	7	IL	2020	Whole muscle meat substitute and methods of obtaining the same	A23J3/22 Working-up of proteins for foodstuffs by texturising	Redefine Meat Ltd.	IL	12	[76]
39	Bom, P.	5	NL	2020	Minced meat analogue	A23J3/225 Texturised simulated foods with high protein content	Unilever Ip Holdings B.V./Conopco, Inc., D/B/A Unilever	NL/US	11	[77]
40	Bonner-Heine, J.	5	US	2020	Plant-based meat alternative compositions	A23L33/105 Plant extracts, their artificial duplicates or their derivatives	Kalamazoo Holdings Inc.	US	7	[78]
41	Breton, O.	3	CH	2020	Method for making meat analogues by extrusion, and suitable extrusion die with a core	A23J3/227 Meat-like textured foods	Société des Produits Nestlé S.A.	CH	5	[79]
42	Demeurisee, J.	1	SE	2020	Meat-analogue composition and process for the preparation thereof	A23L29/10 Foods or foodstuffs containing additives; Preparation or treatment thereof containing emulsifiers	Aak Ab	SE	6	[80]
43	Dikovsky, D.	2	IL	2020	Meat analogue and method of producing the same	A23L13/67 Reformed meat products other than sausages	Redefine Meat Ltd.	IL	3	[81]
44	Dreher, J.	6	DE	2020	Ground meat analogue product	A23J3/14 Vegetable proteins	Société des Produits Nestlé S.A.	CH	8	[82]
45	Ehrlinger, D.J.	3	US	2020	Meat alternative compositions comprising cranberry seed preparations and methods for making same	A23J3/227 Meat-like textured foods	Ocean Spray Cranberries, Inc.	US	1	[83]
46	Frecker, S.	3	US	2020	Color for plant-based meat alternatives	A23L5/43 Addition of dyes or pigments, e.g., in combination with optical brighteners using naturally occurring organic dyes or pigments, their artificial duplicates or their derivatives	Chr Hansen Natural Colors A.S.	DK	1	[84]
47	Gaddipati, S.	2	US	2020	Formed meat analogue product	A23J3/16 Vegetable proteins from soybean	Société des Produits Nestlé S.A.	CH	2	[85]
48	Han, K-S.	3	KR	2020	A method for making sausage analogue using mixed bean protein concentrate	A23L11/07 Soya beans, e.g., oil-extracted soya bean flakes	Sahmyook University Industry-Academic Cooperation Foundation	KR	3	[86]
49	Ingoglia, C.	2	FR	2020	Meat analogs comprising thin flakes for food compositions	A23L13/10 Meat meal or powder; Granules, agglomerates or flakes	Société des Produits Nestlé S.A.	CH	2	[87]
50	Kim, T.W.	2	KR	2020	Meat analogue	A23L13/40 Meat products; Meat meal; Preparation or treatment thereof containing additives	Famenity Co., Ltd.	KR	3	[88]
51	Lee, H.	5	CN	2020	Processing method of vegetable protein substituted meat	A23J3/14 Vegetable proteins	Plant Meat Hangzhou Health Technology Co., Ltd.	CN	7	[89]
52	Legarth, J.H.	2	DK	2020	Meat analogue comprising lab fermented material	A23J3/20 Proteins from microorganisms or unicellular algae	Fermentation Experts A/S	DK	0	[90]
53	Legay, G.	1	FR	2020	Process for preparing a juicy and tender plant-based meat analogue	A23L33/185 Vegetable proteins	Les Nouveaux Fermiers S.A.S.	FR	0	[91]
54	Lundberg, B.M.	2	US	2020	Super-volumetric highly refined cellulose in vegan meat-alternative compositions	A23J3/227 Meat-like textured foods	Fiberstar Inc.	US	5	[92]
55	Mam, H.	1	CN	2020	Vegetarian raw material meat-imitation food formula and processing method thereof	A23L33/16 Inorganic salts, minerals or trace elements	Shanghai Boohee Information Technology Co., Ltd.	CN	6	[93]
56	Mellema, M.	3	NL	2020	Edible composition comprising a structured aqueous phase	A23J3/227 Meat-like textured foods	Unilever N.V./Unilever Plc./Conopco, Inc., D/B/A Unilever	NL/GB/US	13	[94]
57	Nettesheim, F.	4	DK	2020	Plant-based food product	A23J3/227 Meat-like textured foods	Dupont Nutrition Biosciences Aps/DuPont Nutrition USA, Inc.	DK/US	11	[95]
58	Niskakoski, A.K.	7	FI	2020	Method of manufacturing a formed meat-replacement food product and a formed meat-replacement food product comprising a plant-based proteinaceous binder ingredient, a plant-based proteinaceous binder ingredient and a method for manufacturing a plant-based proteinaceous binder ingredient	A23J3/26 Working-up of proteins for foodstuffs by texturising using extrusion or expansion	Gold & Green Foods Oy	FI	0	[96]
59	Park, J.; Kim, J.	12	KR	2020	Process of manufacturing meat substitute food stuff	A23L33/10 Modifying nutritive qualities of foods; Dietetic products; Preparation or treatment thereof using additives	Taekyung Agricultural Products Co., Ltd.	KR	2	[97]
60	Pelloux, C.	3	FR	2020	Plant-based meat analogue prepared by wet extrusion of mixture of plant protein isolates and gluten	A23J3/14 Vegetable proteins	Société des Produits Nestlé S.A.	CH	1	[98]
61	Pibarot, P.	4	CH	2020	Meat analogues and meat analogue extrusion devices and methods	A23J3/227 Meat-like textured foods	Société des Produits Nestlé S.A./Université De Montpellier/Institut National De La Recherche Agronomique	CH/FR/FR	9	[99]
62	Riddle, R.	2	US	2020	Meat analogue product comprising hydrated textured plant protein	A23J3/18 Vegetable proteins from wheat	Société des Produits Nestlé S.A.	CH	11	[100]
63	Ryu, K-H.	2	KR	2020	Method of manufacturing meat analogue patty by extrusion process	A23L33/185 Vegetable proteins	Kongju National University Industry-Academic Cooperation Group	KR	4	[101]
64	Sein, A.	3	NL	2020	Meat alternatives comprising rapeseed protein	A23J1/006 Obtaining protein compositions for foodstuffs; Bulk opening of eggs and separation of yolks from whites from vegetable materials	Dsm Ip Assets B.V.	NL	17	[102]
65	Shamaila, M.	4	DE	2020	Process for manufacturing a formed meat analogue product	A23J3/227 Meat-like textured foods	Société des Produits Nestlé S.A.	CH	6	[103]
66	Smith, T.	4	US	2020	Protein compositions for plant-based food products and methods for making	A23J3/14 Vegetable proteins	Glanbia Nutritionals Limited	IE	8	[104]
67	Spelbrink, R.E.J.	4	NL	2020	Patatin as binder in meat substitutes	A23J3/22 Working-up of proteins for foodstuffs by texturising	Cooperative Avebe UA	NL	2	[105]
68	Sterner, M.H.	2	US	2020	Pulse-based meat substitute	A23P10/25 Agglomeration or granulation by extrusion or by pressing, e.g., through small holes, through sieves or between surfaces	Inland Empire Foods Inc.	US	2	[106]
69	Sui, X.	11	CN	2020	Method for preparing plant-based meat substitute by using plant protein	A23L33/185 Vegetable proteins	Northeast Agricultural University	CN	4	[107]
70	Uno, S.	1	JP	2020	Fat composition	.	NOF Corp.	JP	1	[108]
71	Wang, S.	12	CN	2020	Dried vegetarian meat slice and processing method thereof	A23J3/16 Vegetable proteins from soybean	China Meat Research Centre	CN	11	[109]
72	Wei, X.	1	CN	2020	Vegetable protein artificial meat and preparation method thereof	A23L33/185 Vegetable proteins	Application filed by author	CN	4	[110]
73	Van Leeuwen, N.F.	6	NL	2020	Vegetarian burger	A23J3/225 Texturised simulated foods with high protein content	Unilever Ip Holdings B.V./Conopco, Inc., D/B/A Unilever	NL/US	11	[111]
74	Xiaonan, S.	7	CN	2020	Method for producing structural soybean-based meat analogs by using couette shear flow-pressure tank	A23J1/14 Obtaining protein compositions for foodstuffs; Bulk opening of eggs and separation of yolks from whites from leguminous or other vegetable seeds; from press-cake or oil-bearing seeds	Northeast Agricultural University/Hey Meat Food Technology Co., Ltd.	CN/CN	0	[112]
75	Yoon, J-H.	1	KR	2020	Plant-based meat analogue having improved texture and The Manufacturing method thereof	A23L33/185 Vegetable proteins	Application filed by author	KR	6	[113]
76	Alderton, A.	5	US	2021	Plant-based analog meat compositions and methods of manufacture	A23L11/00 Pulses, i.e., fruits of leguminous plants, for production of food; Products from legumes; Preparation or treatment thereof	Corn Products Development Inc., USA	US	1	[114]
77	Baier, S.	1	US	2021	Plant-based connective tissue analogs	A23J3/227 Meat-like textured foods	Motif Foodworks, Inc.	US	1	[115]
78	Barbarini, A.	1	AR	2021	Meat substitutes produced in plant-based systems and method thereof	A23L19/00 Products from fruits or vegetables; Preparation or treatment thereof	Dr. Eyal Bressler Ltd.	IL	2	[116]
79	Ben-Shitrit, A.	5	CN	2021	Meat analog and its preparation method	A23J3/227 Meat-like textured foods	Redefine Meat Co.,Ltd.	IL	46	[117]
80	Bühler, J.M.	6	NL	2021	Starch addition for improved structure formation in meat analogues	A23J3/227 Meat-like textured foods	Ingredion Germany GmbH/Wageningen Universiteit/Stichting Wageningen Research	DE/NL/NL	0	[118]
81	Cheng, Y.	10	CN	2021	Method for preparing vegetable protein meat by using inulin composite gel as substitute fat	A23J3/16 Vegetable proteins from soybean	Jiangnan University	CN	2	[119]
82	Chuang, J.Ch.	3	JP	2021	Meat substitute composition	A23J3/14 Vegetable proteins	Spiber Inc.	JP	0	[120]
83	Chien, Y-H.	3	NL	2021	Vegetarian hamburger	A23J3/26 Working-up of proteins for foodstuffs by texturising using extrusion or expansion	Dsm Ip Assets B.V.	NL	2	[121]
84	Driesses, M.	2	NL	2021	Meat analogue and process for producing the same	A23J3/14 Vegetable proteins	Unilever Ip Holdings B.V.	NL	0	[122]
85	Felke, B.I.	2	US	2021	Meat substitute product	A23J3/227 Meat-like textured foods	Cargill, Incorporated	US	2	[123]
86	Goto, U.	2	JP	2021	Meat substitute foods, including curd and its manufacturing method	A23J3/00 Working-up of proteins for foodstuffs	Unitec Foods Co. Ltd.	JP	1	[124]
87	Grabinski, D.	2	US	2021	Modification and extrusion of proteins to manufacture moisture texturized protein	A23J3/227 Meat-like textured foods	Nowadays Inc.Pbc.	US	2	[125]
88	Häkämies, A.	2	FI	2021	Method of producing meat analogue food ingredients	A23J3/16 Vegetable proteins from soybean	Solar Foods Oy	FI	0	[126]
89	Han, K-S.	3	KR	2021	Method for preparing functional mixed concentrate protein extract and plant-based meat alternatives and use thereof	A23J3/16 Vegetable proteins from soybean	Samyuk University Industry-Academic Cooperation Foundation	KR	2	[127]
90	Hoon, K.J.	7	KR	2021	Method for preparing artificial ground meat including fiber and artificial ground meat including fiber prepared thereby	A23J3/227 Meat-like textured foods	Intake Co., Ltd./Seoul National University Industry-Academic Cooperation Foundation/Soimaru Co., Ltd.	KR/KR/UK	10	[128]
91	Ito, G.	2	JP	2021	Use of pea starch and its cross linked derivatives to improve the texture of meat products and meat analogues	A23L13/426 Addition of proteins, carbohydrates or fibrous material from vegetable origin other than sugars or sugar alcohols	Roquette Freres S.A.	FR	0	[129]
92	Jeong, G-H.	1	KR	2021	Preparing method for plant-based meat analogue having meat-like texture	A23L33/185 Vegetable proteins	Altist Co., Ltd.	KR	0	[130]
93	Jeongwoo, H.	3	KR	2021	Manufacturing method of meat analogue and meat analogue manufactured thereby	A23J3/227 Meat-like textured foods	Intake Co., Ltd.	KR	1	[131]
94	Jina, H.	2	CN	2021	Process for manufacturing alternative artificial meat	A23L13/72 Tenderised or flavoured meat pieces; Macerating or marinating solutions specially adapted therefore using additives, e.g., by injection of solutions	Fujian Nongke Nongye Development Co., Ltd.	CN	0	[132]
95	Knoch, A.	2	DE	2021	Plant-based meat analogue with muscle-like fibers	A23J3/225 Texturised simulated foods with high protein content	Livekindly Company Switzerland GmbH	CH	1	[133]
96	Lee, S.	2	KR	2021	Method of Making Discrete Frozen Particles of Coconut Fat and a Meat Analogue with the Same	A23D9/04 Working-up	Tonghark Food Co., Ltd./Mosey, Thomas R.	US/KR	0	[134]
97	Liu, J.	4	FI	2021	A meat-replacement product and a method of manufacturing the same	A23J3/26 Working-up of proteins for foodstuffs by texturising using extrusion or expansion	Valio Oy	FI	1	[135]
98	Liu, X.	4	CN	2021	Plant-based meat product and food prepared from same	A23J3/14 Vegetable proteins	Plant Meat Hangzhou Health Technology Co., Ltd.	CN	1	[136]
99	Park, H-S.	2	KR	2021	A method for preparing vegetable fat composition using a physical improvement agent, a method for preparing vegetable meat containing the fat composition, and a method for preparing vegetable meat	A23L29/04 Fatty acids or derivatives	Devotion Food Co., Ltd.	KR	0	[137]
100	Park, H-S.	2	KR	2021	Natural pigment composition for vegetable substitute meat, vegetable substitute meat containing the same, and method for manufacturing the same	A23L5/43 Addition of dyes or pigments, e.g., in combination with optical brighteners using naturally occurring organic dyes or pigments, their artificial duplicates or their derivatives	Devotion Food Co., Ltd.	KR	0	[138]
101	Pasternak, B.T	2	US	2021	Plant-based food products	A23J3/14 Vegetable proteins	Nourish Cult, LLC/True Evolution LLC	US/US	0	[139]
102	Perdana, J.	6	DE	2021	A process for preparing a dehydrated meat-analogue	A23L33/185 Vegetable proteins	Société des Produits Nestlé	CH	6	[140]
103	Perera, Ch.	1	US	2021	Plant-based meat analog	A23J3/227 Meat-like textured foods	Roquette Freres S.A.	FR	1	[141]
104	Pyett, S.Ch.	2	NL	2021	Meat or fish substitute, and method for preparing the same	A23L29/20 Foods or foodstuffs containing additives; Preparation or treatment thereof containing gelling or thickening agents	Stichting Wageningen Res	NL	0	[142]
105	Sato, T.	2	JP	2021	Method for manufacturing meat-like processed food product	A23L13/426 Addition of proteins, carbohydrates or fibrous material from vegetable origin other than sugars or sugar alcohols	Fuji Oil Holdings Inc.	JP	3	[143]
106	Schlebusch, J.P.	1	DE	2021	The process for production of a meat analogue, and meat analogue prepared thereby	A23J3/26 Working-up of proteins for foodstuffs by texturising using extrusion or expansion	Mars, Incorporated	US	0	[144]
107	Seoyoung, P.	1	KR	2021	Manufacturing method of alternative meat having marbling	A23L33/185 Vegetable proteins	SY Solutions Co., Ltd.	TH	0	[145]
108	Srichuwong, S.	6	DE	2021	Meat and seafood analogue products	A23J3/14 Vegetable proteins	Bk GiuliniGmbh/Plantible Foods Inc.	DE/US	0	[146]
109	Sung, Y.	1	JP	2021	Pseudo-meat food product and method for producing pseudo-meat food product	A23J3/14 Vegetable proteins	Banseisha Co., Ltd.	JP	1	[147]
110	Titmus, M.	2	GB	2021	A cooked meat substitute and method of preparing same	A23J3/227 Meat-like textured foods	Kerry Luxembourg SARL	LU	0	[148]
111	Wang, X.	1	CN	2021	Vegetarian meat processing technology	A23J3/16 Vegetable proteins from soybean	Shuangfeng Weilong Food Co., Ltd.	CN	1	[149]
112	Van Gurp, M.J.C.	9	NL	2021	Functional potato protein compositions with reduced enzymatic activity	A23J1/006 Obtaining protein compositions for foodstuffs; Bulk opening of eggs and separation of yolks from whites from vegetable materials	Duynie Holding B.V.	NL	0	[150]
113	Verbeeck, S	3	BE	2021	Meat substitute	A23J3/227 Meat-like textured foods	Fuji Oil Europe	BE	0	[151]
114	Zhang, W.	5	CN	2021	High-moisture plant-based substitute meat and preparation method thereof	A23J3/16 Vegetable proteins from soybean	Sutuo Technology Shenzhen Co.,Ltd.	CN	4	[152]
115	Zotter, B.A.	2	US	2021	Food products resembling whole muscle meat and seafood	A23J3/225 Texturised simulated foods with high protein content	Umaro Foods Inc.	US	7	[153]
116	Acosta Fernández, R.A.	2	CO	2022	Animal fat tissue substitutes for meat products and alternatives and preparation method thereof	A23D7/0053 Compositions other than spreads	Team Foods Colombia S.A.	CO	0	[154]
117	Amiel, D	1	IL	2022	Instant vegan meat analog granulated powder and methods of making same	A23J3/14 Vegetable proteins	Mixoy Israel MI Ltd.	IL	0	[155]
118	Bezenek, T.	1	AT	2022	Method for producing a layered vegetarian or vegan food item or meat substitute product	A23J3/225 Texturised simulated foods with high protein content	Application filed by author	AT	1	[156]
119	Breton, O.	11	CH	2022	Plant-based meat and fish analog products	A23P30/20 Extruding	Société des Produits Nestlé S.A.	CH	1	[157]
120	Brouwer, F.	2	NL	2022	Coated meat or fish substitute	A23L27/215 Synthetic spices, flavouring agents or condiments containing amino acids heated in the presence of reducing sugars, e.g., Maillard’s non-enzymatic browning	Dsm Ip Assets B.V.	NL	0	[158]
121	Budolfsen, G.	2	DK	2022	Method for producing a meat analogue product	A23J3/346 Working-up of proteins for foodstuffs by hydrolysis using chemical agents using enzymes of vegetable protein	Novozymes A/S	DK	1	[159]
122	De Lange, L.	2	NL	2022	Meat analogue product	A23L33/115 Fatty acids or derivatives thereof; Fats or oils	Dsm Ip Assets B.V.	NL	0	[160]
123	Demeurisse, J.	1	SE	2022	Meat-analogue composition comprising saturated fatty acids of stearic and lauric acid residues	A23D7/0053 Compositions other than spreads	Aak Ab	SE	2	[161]
124	Evans, C.C.	3	US	2022	Plant-based meat alternative product with a meat-like color appearance	A23J3/14 Vegetable proteins	Société des Produits Nestlé S.A.	CH	1	[162]
125	Evans, C.C.	2	US	2022	Meat analogue product	A23J3/14 Vegetable proteins	Société des Produits Nestlé S.A.	CH	4	[163]
126	Garuda, L.	4	IL	2022	Edible plant-based protein composition	A23L33/18 Peptides; Protein hydrolysates	Meala Foodtech Ltd.	IL	0	[164]
127	Ge, Y	5	CN	2022	Meat substitute product	A23J3/227 Meat-like textured foods	Cargill, Incorporated	US	0	[165]
128	Griffin, W.B.	3	US	2022	Meat analogue products comprising modified starch	A23L35/10 Emulsified foodstuffs	Cargill, Incorporated	US	0	[166]
129	Halevi, O.	2	IL	2022	Whole-muscle meat analogues with fluid accommodating spaces and method of producing the same	A23J3/227 Meat-like textured foods	Redefine Meat Ltd.	IL	1	[167]
130	Hashimoto, S.	2	JP	2022	Textured plant protein material and method for producing same	A23J3/16 Vegetable proteins from soybean	Fuji Oil Holdings Inc.	JP	0	[168]
131	Jihyeong, S-G.	4	KR	2022	Manufacturing method of vegetable meat using fruit	A23L33/185 Vegetable proteins	Chong Kun Dang Health Co., Ltd.	KR	0	[169]
132	Kang, B.	5	KR	2022	Meat-product substitute material and dumplings	A23L29/262 Cellulose; Derivatives thereof, e.g., ethers	CJ Cheil Jedang Corp.	KR	0	[170]
133	Kang, K.H.	2	KR	2022	Method for making vegan dumplings	A23P20/20 Making of laminated, multi-layered, stuffed or hollow foodstuffs, e.g., by wrapping in preformed edible dough sheets or in edible food containers	Hyosung Food Agricultural Cooperative Association	KR	0	[171]
134	Kitazawa, D.	3	JP	2022	Formulation for producing plant-based-protein-containing food product	A23J3/227 Meat-like textured foods	Ajinomoto Co., Inc.	JP	0	[172]
135	Kohli, N.	4	US	2022	Non-heme protein pigments for meat substitute compositions	A23L5/43 Addition of dyes or pigments, e.g., in combination with optical brighteners using naturally occurring organic dyes or pigments, their artificial duplicates or their derivatives	Cargill, Incorporated	US	0	[173]
136	Lee, J.	3	US	2022	Methods of binding ingredients of meat analog products	A23J3/16 Vegetable proteins from soybean	Archer-Daniels-Midland Company	US	1	[174]
137	Ludovici, K.	2	DE	2022	Raw material composition and processing methods	A23L33/185 Vegetable proteins	Endori Food & Co. Kg GmbH	DE	0	[175]
138	Machen, M.A.	4	US	2022	Meat substitute products free of methylcellulose	A23L29/244 Foods or foodstuffs containing additives; Preparation or treatment thereof containing gelling or thickening agents of vegetable origin from corms, tubers or roots, e.g., glucomannan	Cargill, Incorporated	US	0	[176]
139	Maj, H.	4	SI	2022	Method of producing a meat analogue	A23J3/26 Working-up of proteins for foodstuffs by texturising using extrusion or expansion	Bevo, Biotehnološke Rešitve D.O.O.	SI	1	[177]
140	Malmros, H.	6	SE	2022	Meat-analogue composition comprising an interesterified blend of vegetable oil and fully hydrogenated vegetable oil	A23J3/227 Meat-like textured foods	AAK AB	SE	1	[178]
141	Matsuno, R.	1	JP	2022	Fatty mass composition and meat alternative	A23D7/0053 Compositions other than spreads	Fujifilm Co., Ltd.	JP	1	[179]
142	Meng, Z.	2	CN	2022	Vegetable meat substitute fat based on peanut oil body and preparation method and application thereof	A23D7/0053 Compositions other than spreads	Jiangnan University	CN	0	[180]
143	Minamikawa, T.	1	JP	2022	Method for producing meat-like processed food	A23J3/14 Vegetable proteins	Minami Songyo Co., Ltd.	JP	0	[181]
144	Mohanan, A.	8	CA	2022	Dairy and meat analogues containing euglena-derived components	A23D7/0053 Compositions other than spreads	Noblegen Inc.	CA	0	[182]
145	Qin, X.	8	CN	2022	Preparation method of plant-based meat substitute and plant-based meat substitute	A23J3/227 Meat-like textured foods	Angel Yeast Co., Ltd.	CN	1	[183]
146	Park, H.J.;	3	KR	2022	Method for manufacturing plant-based meat with artifical muscle fiber inserted	A23P30/20 Extruding	Bippeco	KR	1	[184]
147	Pinol, S.H.	5	ES	2022	Protein gel composition providing improved texture to a meat analog product	A23J3/14 Vegetable proteins	Current Foods, Inc.	US	0	[185]
148	Sakaguchi, R.	2	JP	2022	Meat substitutional food and production method of the same	.	Showa Sangyo Co., Ltd.	JP	0	[186]
149	Seonghee, Ch.	5	KR	2022	Alternative Meat Production Method Using Soybeans	A23L33/185 Vegetable proteins	Sunmoon University Industry-Academic Cooperation Foundation	KR	0	[187]
150	Solorio, S.	8	US	2022	Extruded food product comprising plant protein and hydrocolloid	A23L29/256 Foods or foodstuffs containing additives; Preparation or treatment thereof containing gelling or thickening agents of vegetable origin from seaweeds, e.g., alginates, agar or carrageenan	Dupont Nutrition Biosciences Aps/DuPont Nutrition USA, Inc./Solae Llc.	US/US/US	0	[188]
151	Song, M.	1	KR	2022	Gluten-free meat alternative plant-based meat and manufacturing method thereof	A23J3/227 Meat-like textured foods	FutureX Co., Ltd.	KR	1	[189]
152	Stanišic, N.	4	NL	2022	Patatin-emulsified binder	A23L33/185 Vegetable proteins	CoöperatieKoninklijkeAvebe U.A.	NL	0	[190]
153	Stidham, L.	3	US	2022	Process for improving flavor of meat analogs	A23L33/185 Vegetable proteins	Givaudan S.A.	CH	0	[191]
154	Sui, X.	7	CN	2022	Method for preparing spicy meat sausage by using soybean protein concentrate-sodium alginate mixed system and pea protein	A23J3/16 Vegetable proteins from soybean	Northeast Agricultural University	CN	0	[192]
155	Tomsov, A.	5	IL	2022	Food analogues preparation method and products	A23L33/185 Vegetable proteins	Redefine Meat Ltd.	IL	0	[193]
156	Tsukamoto, N.	1	JP	2022	Raw meat-like meat alternative and method for producing raw meat-like meat alternative	A23J3/227 Meat-like textured foods	Fujifilm Corp.	JP	0	[194]
157	Ullmann, R.	9	DE	2022	A composition of a dehydrated meat analogue product	A23J3/14 Vegetable proteins	Société des Produits Nestlé S.A.	CH	0	[195]
158	Uzunalioglu, D.	2	US	2022	Meat alternative formulation	A23J3/14 Vegetable proteins	Motif Foodworks, Inc.	US	2	[196]
159	Williams, C.	5	GB	2022	Meat analogues	A23L29/015 Inorganic compounds	Plant Meat Ltd.	GB	0	[197]
160	Ya, R.;	3	NL	2022	Vegetarian sausages	A23L29/269 Foods or foodstuffs containing additives; Preparation or treatment thereof containing gelling or thickening agents of microbial origin, e.g., xanthan or dextran	Dsm Ip Assets B.V.	NL	2	[198]
161	Aono, N.	2	JP	2023	Protein food material and alternative molded meat	A23J3/00 Working-up of proteins for foodstuffs	Fujifilm Co., Ltd.	JP	0	[199]
162	Baier, S.K.	4	US	2023	Marbled meat analog and methods of making	A23L29/262 Cellulose; Derivatives thereof, e.g., ethers	Motif Foodworks, Inc.	US	0	[200]
163	Cohen-Jonatha, N.A.	2	IL	2023	Process for manufacturing vegan meat and components thereof	A23J3/227 Meat-like textured foods	Limeatless Food Ltd.	IL	0	[201]
164	Coiffier, J.	2	US	2023	Plant- or fungi-based layered meat analog and methods of making the same	A23J3/14 Vegetable proteins	Terramino, Inc.	US	0	[202]
165	Cros, A.	4	CH	2023	Fat analogue for use in a meat analogue product	A23D7/0053 Compositions other than spreads	Société des Produits Nestlé S.A.	CH	0	[203]
166	Dzikovsky, D.	2	IL	2023	Meat analogue and method of producing the same	A23J3/227 Meat-like textured foods	Redefine Meat Ltd.	IL	3	[204]
167	Ellis, Ch.,M.	1	US	2023	Instant texturized meat alternative	A23J3/227 Meat-like textured foods	Steuben Foods, Inc.	US	0	[205]
168	English, A.R.	2	US	2023	Alternative protein crumbles	A23L13/60 Comminuted or emulsified meat products, e.g., sausages; Reformed meat from comminuted meat product	Cargill, Incorporated	US	0	[206]
169	Garg, P.	5	IN	2023	Process for preparing a formed meat analogue product	A23J3/14 Vegetable proteins	Société des Produits Nestlé S.A.	CH	0	[207]
170	Hernandez, P.Z.	3	BE	2023	Plant-based ground and formed meat alternatives	A23L29/212 Starch; Modified starch; Starch derivatives, e.g., esters or ethers	Cargill, Incorporated	US	0	[208]
171	Hossen, M.	5	US	2023	Sliced meat analogues and production thereof	A23J3/227 Meat-like textured foods	Kellogg Company	US	0	[209]
172	Jeradechachai, T.	4	US	2023	Plant-based meat alternative compositions for foodservice and preparation methods thereof	A23J3/14 Vegetable proteins	Mgpi Processing, Inc.	US	0	[210]
173	Nixon, J.	2	US	2023	Food products including carotenoids for improved coloring and methods of making the same	A23L5/44 Addition of dyes or pigments, e.g., in combination with optical brighteners using naturally occurring organic dyes or pigments, their artificial duplicates or their derivatives using carotenoids or xanthophylls	Terramino, Inc.	US	0	[211]
174	Ong, S.	1	SG	2023	Method for scalable production of meat chunks	A23J3/227 Meat-like textured foods	Ants Innovate Pte. Ltd.	SG	4	[212]
175	Santagiuliana, M.	5	NL	2023	Meat analogue and process to produce the same	A23J3/26 Working-up of proteins for foodstuffs by texturising using extrusion or expansion	Unilever Ip Holdings B.V./Conopco, Inc., D/B/A Unilever	NL/US	0	[213]
176	Sein, A.	2	NL	2023	Texturized vegetable protein	A23J3/26 Working-up of proteins for foodstuffs by texturising using extrusion or expansion	Dsm Ip Assets B.V.	NL	0	[214]
177	Schmelzeisen, D.	2	DE	2023	Fibre composite of multi-component filaments for emulating meat	A23L29/256 Foods or foodstuffs containing additives; Preparation or treatment thereof containing gelling or thickening agents of vegetable origin from seaweeds, e.g., alginates, agar or carrageenan	Project Eaden Gmbh	DE	1	[215]
178	Singh, J.	6	NZ	2023	Process for preparing hybrid meat analogue	A23J3/227 Meat-like textured foods	Massey University	NZ	0	[216]
179	Takinami, T.	4	JP	2023	Meat alternative processed food, method for producing same, method for improving texture thereof, and texture improver for meat alternative processed food	A23J3/00 Working-up of proteins for foodstuffs	Nichirei Foods Co., Ltd.	JP	0	[217]
180	Tsukamoto, N.	1	JP	2023	Vegetable protein binder, chunk meat-like meat alternative, and method of producing chunk meat-like meat alternative	A23J3/227 Meat-like textured foods	Fujifilm Corp.	JP	0	[218]
181	Tsukamoto, N.	2	JP	2023	Method of producing chunk meat-like meat alternative and chunk meat-like meat alternative	A23J3/227 Meat-like textured foods	Fujifilm Corp.	JP	1	[219]
182	Weis, A.	6	SE	2023	Meat-analogue composition	A23J3/227 Meat-like textured foods	Aak Ab	SE	0	[220]
183	Yamada, S.	3	JP	2023	Meat substitutional food product containing defatted soybean flour	.	Showa Sangyo Co., Ltd.	JP	0	[221]

**Table A2:** The total number of patents referring to plant-based meat analogues (PBMA) developed in the period 2014–2023 by a varying number of authors, including the affiliation of the first author and the affiliation of applicants. The different letters mean statistically significant differences. Country name code abbreviations as in Figure 3.

		Years										Mean (±SD)	The Value of H Kruskal–Wallis Test, *p* Value
		2014	2015	2016	2017	2018	2019	2020	2021	2022	2023		
Number of authors	1	3	1	0	2	2	0	6	6	7	3	2.82 (±2.48) abc	48.76, *p* < 0.01
	2	2	2	1	0	4	3	9	16	12	8	5.36 (±5.16) ab	
	3	0	1	0	1	5	1	7	5	7	2	2.91 (±2.66) abc	
	4	0	0	0	1	0	3	5	2	6	4	2.27 (±2.24) abc	
	5	0	1	1	1	0	0	3	3	6	3	2.09 (±2.07) abc	
	6	0	0	0	0	0	1	2	3	1	2	1.36 (±1.86) abc	
	7	0	0	0	0	0	0	3	1	1	0	1.09 (±2.17) abc	
	8	0	1	0	0	0	0	0	0	2	0	1.00 (±2.41) c	
	9	0	1	0	0	0	0	0	1	1	0	1.09 (±2.66) c	
	10	0	0	0	0	0	0	0	1	0	0	1.00 (±3.00) c	
	11	0	0	0	0	0	0	1	0	1	0	1,18 (±3.28) c	
	12	0	0	0	0	0	0	1	0	0	0	1,18 (±3.60) c	
Firstauthor affiliation	US	0	2	0	2	1	0	8	7	9	7	3.60 (±3.69)	66.92, *p* < 0.01
	KR	0	0	0	0	4	1	4	8	6	0	2.30 (±2.98)	
	JP	1	1	0	1	1	1	1	5	6	5	2.20 (±2.20)	
	CN	2	0	0	0	0	1	6	6	4	0	1.90 (±2.51)	
	NL	0	0	0	0	2	1	5	5	4	2	1.90 (±2.08)	
	DE	1	1	0	0	0	0	2	4	2	1	1.10 (±1.29)	
	IL	0	0	0	1	0	0	3	0	4	1	0.90 (±1.45)	
	CH	0	0	1	0	0	1	2	0	1	1	0.60 (±0.70)	
	FI	0	1	0	0	1	1	1	2	0	0	0.60 (±0.70)	
	BE	1	0	0	1	0	0	0	1	0	1	0.40 (±0.52)	
	DK	0	0	0	0	1	0	2	0	1	0	0.40 (±0.70)	
	SE	0	0	0	0	0	0	1	0	2	1	0.40 (±0.70)	
	FR	0	0	0	0	0	0	3	0	0	0	0.30 (±0.95)	
	GB	0	0	0	0	1	0	0	1	1	0	0.30 (±0.48)	
	CA	0	0	0	0	0	1	0	0	1	0	0.20 (±0.42)	
	IN	0	1	0	0	0	0	0	0	0	1	0.20 (±0.42)	
	NZ	0	0	1	0	0	0	0	0	0	1	0.20 (±0.42)	
	AR	0	0	0	0	0	0	0	1	0	0	0.10 (±0.32)	
	AT	0	0	0	0	0	0	0	0	1	0	0.10 (±0.32)	
	CO	0	0	0	0	0	0	0	0	1	0	0.10 (±0.32)	
	ES	0	0	0	0	0	0	0	0	1	0	0.10 (±0.32)	
	GR	0	0	0	0	0	1	0	0	0	0	0.10 (±0.32)	
	PL	0	1	0	0	0	0	0	0	0	0	0.10 (±0.32)	
	SG	0	0	0	0	0	0	0	0	0	1	0.10 (±0.32)	
	SI	0	0	0	0	0	0	0	0	1	0	0.10 (±0.32)	
Applicant affiliation	US	0	2	0	2	4	0	8	10	10	9	4.50 (±4.30)	71.59, *p* < 0.01
	KR	0	0	0	0	4	1	4	7	7	0	2.30 (±2.95)	
	JP	1	1	0	1	2	1	1	4	6	5	2.20 (±2.04)	
	CH	0	1	1	0	0	1	8	2	5	2	2.00 (±2.58)	
	NL	0	0	0	0	1	1	6	6	4	2	2.00 (±2.45)	
	CN	2	0	0	0	0	1	7	5	3	0	1.80 (±2.49)	
	IL	0	0	0	1	0	0	3	2	4	1	1.10 (±1.45)	
	DE	1	1	0	0	0	0	0	2	1	1	0.60 (±0.70)	
	FI	0	1	0	0	1	1	1	2	0	0	0.60 (±0.70)	
	DK	0	0	0	0	1	0	3	0	1	0	0.50 (±0.97)	
	FR	0	0	0	0	0	0	3	2	0	0	0.50 (±1.08)	
	GB	0	0	0	0	2	0	1	1	1	0	0.50 (±0.71)	
	BE	1	0	0	1	0	0	0	1	0	0	0.30 (±0.48)	
	SE	0	0	0	0	0	0	1	0	2	1	0.40 (±0.70)	
	CA	0	0	0	0	0	1	0	0	1	0	0.20 (±0.42)	
	NZ	0	0	1	0	0	0	0	0	0	1	0.20 (±0.42)	
	AT	0	0	0	0	0	0	0	0	1	0	0.10 (±0.32)	
	CO	0	0	0	0	0	0	0	0	1	0	0.10 (±0.32)	
	GR	0	0	0	0	0	1	0	0	0	0	0.10 (±0.32)	
	IE	0	0	0	0	0	0	1	0	0	0	0.10 (±0.32)	
	LU	0	0	0	0	0	0	0	1	0	0	0.10 (±0.32)	
	PL	0	1	0	0	0	0	0	0	0	0	0.10 (±0.32)	
	SG	0	0	0	0	0	0	0	0	0	1	0.10 (±0.32)	
	SI	0	0	0	0	0	0	0	0	1	0	0.10 (±0.32)	
	TH	0	0	0	0	0	0	0	1	0	0	0.10 (±0.32)	

**Table A3:** Plant taxa characteristic according to Fern (2024) [222]. Lifespan: A—annual, P—perennial; Abbreviations: Life form: H—herbaceous plant, S—shrub, T—tree. The empty cells mean a lack of data.

Family	Taxon		Lifespan	Life Form	Edibility Rating
	Latin Name	Selected Common Names			
*Actinidiaceae*	*Actinidia* sp. Lindl.	Kiwifruit			
*Adoxaceae*	*Sambucus sp.* L.	Elder, elderflower, elderberry			
*Amaranthaceae*	*Amaranthus* sp. L.	Amaranth			
	*Beta vulgaris* L.	Beet, beetroot	Biennial	H	5
	*Beta vulgaris* L. *subsp. vulgaris*	Sugar beet	Biennial	H	5
	*Celosia* sp. L.	Cockscomb			
	*Chenopodium pallidicaule* Aellen	Kaniwa, cañihua	Annual	H	3
	*Chenopodium quinoa* Willd.	Quinoa	Annual	H	5
	*Spinacia oleracea* L.	Spinach	Annual	H	3
*Amaryllidaceae*	*Allium cepa* L.	Onion	Perennial	H	5
*Anacardiaceae*	*Anacardium occidentale* L.	Cashew	Perennial	S or T	5
	*Mangifera indica L.*	Mango	Perennial	T	5
	*Pistacia vera L.*	Pistachi, pistachio	Perennial	T	4
*Annonaceae*	*Annona cherimola* Mill.	Cherimoya	Perennial	T	5
*Apiaceae*	*Apium graveolens* L.	Celery, Wild celery	Biennial	H	3
	*Arracacia xanthorrhiza* Bancr.	Arracacha, racacha	Perennial	H	4
	*Coriandrum sativum* L.	Coriander	Annual	H	4
	*Daucus carota subsp. sativus* (Hoffm.) Schübl. & G artens	Carrot	Biennial	H	5
	*Petroselinum crispim* (Mill.) Fuss	Parsley, garden parsley			
*Araceae*	*Amorphophallus konjac* K. Koch	Konjac, koniak, Devil’s tongue	Perennial	H	4
	*Colocasia esculenta* (L.) Schott	Taro	Perennial	H	4
*Arecaceae*	*Attalea speciosa* Mart.	Babassu	Perennial	T	4
	*Cocos nucifera* L.	Coconut	Perennial	T	5
	*Euterpe oleracea* Mart.	Acai	Perennial	T	5
*Asparagaceae*	*Agave sisalana* Perrine	Sisal	Perennial	H	2
	*Asparagus officinalis* L.	Asparagus	Perennial	H	4
*Asteraceae*	*Artemisia vulgaris* L.	Mugwort	Perennial	H	2
	*Calendula officinalis* L.	Calendula, Pot marigold	Annual	H	3
	*Carthamus tinctorium* L.	Safflower, benibana	Annual	H	4
	*Cynara cardunculus* var. *scolymus* L.	Artichoke, Globe artichoke	Perennial	H	3
	*Guizotia abyssinica* (L. f.) *Cass*	Ramtil, Niger seed	Annual	H	3
	*Helianthus annuus* L.	Sunflower	Annual	H	5
	*Tussilago farfara* L.	Coltsfoot	Perennial	H	3
*Betulaceae*	*Corylus avellana* L.	Hazelnut, hazel	Perennial	S or T	5
*Bixaceae*	*Bixa orellana* L.	Annatto	Perennial	S or T	3
*Boraginaceae*	*Borago officinalis* L.	Borago, Borage, starflower	Annual or biennial	H	3
*Brassicaceae*	*Brassica carinata* A.Braun	Carinata, Ethiopian rape, Ethiopian mustard, Abyssinian Cabbage	Annual, biennial or perennial	H	4
	*Brassica Juncea* (L.)*Czern.*	Brown mustard, Chinese mustard, Indian mustard, Korean green mustard, leaf mustard, oriental mustard, vegetable mustard	Annual or biennial	H	4
	*Brassica napus* L.	Canola, rapeseed, rape, colza	Annual or biennial	H	3
	*Brassica oleracea* L.	Kale, leaf cabbage, wild cabbage	Biennial or perennial	H	3
	*Brassica oleracea* var. *Italica* Plenck	Broccoli	Biennial	H	4
	*Brassica oleracea* var. *botrytis* L.	Cauliflower	Biennial	H	3
	*Brassica oleracea* var. *gongylodes* L.	Kohlarbi	Biennial	H	3
	*Brassica rapa* (L.) *Koch.*	Field mustard, turnip	Biennial	H	4
	*Camelina sativa* (L.) Crantz	False flax, camelina, gold of pleasure, wild flax, linseed dodder, German sesame, Siberian oilseed	Annual	H	3
	*Moringa oleifera* Lam.	Horseradish Tree, moringa	Perennial	S or T	4
	*Raphanus sativus* L.	Radish	Annual	H	4
	*Rhamphospermum nigrum* L. Al-Shehbaz	Black mustard	Annual	H	3
	*Sinapis alba* L.	White mustard	Annual	H	3
*Bromeliaceae*	*Ananas comosus* (L.) Merr.	Pineapple	Perennial	H	5
*Campanulaceae*	*Codonopsis lanceolata* (Siebold & Zucc.) Trautv.	Deodeok, todok, lance asiabell	Perennial	H	3
*Cannabaceae*	*Cannabis sativa* L.	Hemp	Annual	H	4
*Caryocaraceae*	*Caryocar brasiliense* Cambess.	Pequi	Perennial	S or T	4
*Clusiaceae*	*Allanblackia floribunda* Oliv.	Tallow tree, allablackia, vegetable tallow	Perennial	T	3
	*Garcinia indica* Choisy	Kokum, goa butter	Perennial	T	3
*Combretaceae*	*Terminalia anogeissiana* Gere &Boatwr.	Axlewood, bakli, baajhi, dhau, dhawa, dhawra, dhaora	Perennial	T	2
*Convolvulaceae*	*Ipomoea batatas* (L.) Lam.	Sweet potato	Perennial	H	5
*Cucurbitaceae*	*Citrullus lanatus* (Thunb.) Matsum. & Nakai	Water melon	Annual	H	4
	*Cucurbita foetidissima* Kunth	Buffalo gourd	Perennial	H	3
	*Cucurbita pepo* L.	Pumpkin	Annual	H	4
	*Cucurbita pepo* var. *cylindrica* L.	Zucchini			
*Dipterocarpaceae*	*Shorearobusta* Roth	sal tree, sāla, shala, sakhua, sarai	Perennial	T	4
	*Shoreastenoptera Burck.*	Borneo tallow tree, illipe	Perennial	T	3
*Elaeagnaceae*	*Hippophaerhamnoides* L.	Sea buckhorn	Perennial	S or T	5
*Ericaceae*	*Oxycoccus* sp. Hill.	Cranberry			
	*Vaccinium* sect. *cyanococcus* Rydb.	Blueberry			
*Euphorbiaceae*	*Jatropha* sp. L.	Jatropha			
	*Manihot esculenta* Crantz.	Tapioca, cassava	Perennial	S	5
	*Plukenetia volubilis* L.	Sacha ichni	Perennial	S	3
	*Ricinus communis* L.	Castor, castor Bean, castor-oil plant	Perennial	H	1
	*Vernicia fordii* (Hemsl.) Airy Shaw	Tung tree, tungoil tree, kalo nut tree, China wood-oil tree	Perennial	T	1
*Fabaceae*	*Acacia* sp. Mill.	Wattle			
	*Arachis hypogaea* L.	Peanut, groundnut, goober, goober pea, pindar, monkey nut, arachis	Annual	H	4
	*Astragalus gummifer* Labill.	tragacanth, gum tragacanth milkvetch	Perennial	S	3
	*Cajanus cajan* (L.) Millsp	Pigeon pea	Perennial	S	4
	*Cassia* sp. L.	Cassia			
	*Ceratonia siliqua* L.	Carob, locust bean	Perennial	S or T	3
	*Cicer arietinum* L.	Chickpea, channa, chana, garbanzo	Annual	H	4
	*Cyamopsis tetragonoloba*(L.) Taub	Guar, cluster bean	Annual, biennial or perennial	H	4
	*Glycine max* L. Merr.	Soybean	Annual	H	4
	*Lablab purpureus* (L.) Sweet	Hyacinth Bean	Perennial	H	4
	*Lathyrus oleraceus* Lam.	Pea	Annual	H	4
	*Lupinus* sp. L.	Lupine, lupin			
	*Medicago sativa* L.	Alfalfa	Perennial	H	4
	*Phaseolus lunatus* L.	Lima bean	Annual or perennial	H	4
	*Phaseolus vulgaris* L	Kidney bean, French bean	Annual or perennial	H	5
	*Prosopis* sp. L.	Mesquite			
	*Pueraria montana* (Lour.) Merr.	Kudzu, mealy kudzu	Perennial	H	4
	*Tamarindus indica* L.	Tamarind	Perennial	T	4
	*Tara* sp. Molina	Tara			
	*Trifolium* sp. L.	Clover			
	*Trigonella foenum-graecum L.*	Fenugreek	Annual	H	4
	*Vicia sp.*	Vetch			
	*Vicia faba* L.	Fava bean, broad bean, horse bean	Annual	H	4
	*Vicia lens* (L.) Coss. & Germ.	Lentil	Annual	H	5
	*Vigna angularis* (Willd.) Ohwi & H. Ohashi	Red bean, adzuki bean	Annual	H	4
	*Vigna radiata* (L.) R. Wilczek	Mung bean	Annual	H	4
	*Vigna subterranea* (L.) Verdc.	Bambara bean	Annual	H	3
	*Vigna unguiculata subsp. stenophylla* (Harv.) Maréchal, Mascherpa&Stainier	Cowpea	Perennial	H	Inconsiderable
	*Vigna unguiculata* subsp. *unguiculata* (L.) Walp.	Black-eyed bean, Black-eyed pea	Annual	H	4
*Fagaceae*	*Fagus* sp. L.	Beech			
*Grossulariaceae*	*Ribes nigrum* L.	Black currant	Perennial	S	5
	*Ribes rubrum* L.	Red currant	Perennial	S	4
	*Ribes uva-crispa* L.	Gooseberry	Perennial	S	5
*Juglandaceae*	*Carya illinoinensis* (Wangenh.) K. Koch	Pecan	Perennial	T	4
	*Juglans regia* L.	Walnut	Perennial	T	4
*Lamiaceae*	*Lallemantia royleana* Benth. in Wall.	Lallemantia, balangu			
	*Perilla frutescens* (L.) Britton	Perilla, egoma, shiso	Annual or biennial or perennial	H	4
	*Salvia hispanica* L.	Mexican chia	Annual	H	3
*Lauraceae*	*Persea americana* Mill.	Avocado, avocado pear	Perennial	T	5
*Lecythidaceae*	*Bertholletia excelsa* Humb. &Bonpl.	Brasil nut, Brazil nut	Perennial	T	5
*Liliaceae*	*Erythronium japonicum* Decne.	Katakuri	Perennial	H	3
*Limnanthaceae*	*Limnanthes* sp. R.Br.	Meadowfoam			
*Linaceae*	*Linum usitatissimum* L.	Flax, common flax, lineseed	Annual	H	4
	*Punica granatum* L.	Pomegranate	Perennial	S or T	4
*Malvaceae*	*Abelmoschus esculentus* (L.) Moench	Okra	Annual	H	4
	*Ceiba pentandra* (L.) Gaertn.	Kapok, kapok tree	Perennial	T	3
	*Gossypium* sp. L.	Cotton			
	*Hibiscus* sp. L.	Hibiscus			
	*Hibiscus cannabinus* L.	Kenaf	Annual, biennial or perennial	H	4
	*Malva* sp. L.	Mallow			
	*Sterculia urens* Roxb.	Kulu, Indian tragacanth, karaya, gum karaya, katira, sterculia gum, kateera gum	Perennial	T	3
	*Theobroma cacao* L.	Cocoa, cacao	Perennial	T	5
*Marantaceae*	*Maranta arundinacea* L.	Arrowroot, maranta, West Indian arrowroot, obedience plant, Bermuda arrowroot, araru, araruta, Ararat, hulankeeriya	Perennial	H	4
*Moraceae*	*Artocarpus altilis* (Parkinson) Fosberg	Breadfruit	Perennial	T	5
	*Artocarpus camansi* Blanco	Breadnut	Perennial	T	4
	*Artocarpus heterophyllus* Lam.	Jackfruit	Perennial	T	5
	*Morus* sp. L.	Mulberry			
*Musaceae*	*Musa* sp. L.	Banana			
	*Musa textilis* Née	Abaca	Perennial	H	None known
*Myrtaceae*	*Psidium guajava* L.	Common guava, yellow guava, lemon guava, apple guana, guava	Perennial	S or T	5
*Nelumbonaceae*	*Nelumbo nucifera* Gaertn.	Lotus, Indian lotus, sacred water lotus	Perennial	H	4
*Oleaceae*	*Olea europaea* L.	Olive	Perennial	T	4
*Onagraceae*	*Oenothera biennis* L.	Evening primrose	Biennial	H	3
*Oxalidaceae*	*Oxalis tuberosa* Molina	Oca, uqa, yam	Perennial	H	5
*Papaveraceae*	*Papaver* sp. L.	Poppy			
*Pedaliaceae*	*Sesamum indicum* L.	Sesame	Annual	H	4
*Pinaceae*	*Pinus* sp. L.	Pine			
*Plantaginaceae*	*Plantago major* L.	Broadleaf plantain, common plantain, white man’s footprint, waybread, greater plantain	Perennial	H	2
	*Plantago ovata* Forsk	Psyllium, blond psyllium	Annual	H	2
*Poaceae*	*Avena sativa* L.	Oats	Annual	H	4
	*Cenchrus americanus* (L.) Morrone	Millet, pearl millet	Annual	H	3
	*Digitariaexilis* (Kippist) Stapf	Fonio, fonio millet	Annual	H	3
	*Eragrostis tef* (Zucc.) Trotter	Teff, tef	Annual	H	3
	*Hordeum vulgare* L.	Barley	Annual	H	4
	*Oryza sativa* L.	Rice	Annual or perennial	H	5
	*Secale cereale* L.	Rye	Annual	H	4
	*Saccharum* sp. L.	Sugar cane, sugarcane			
	*Sorghum bicolor* L. Moench	Sorghum, great millet, broomcorn, guinea corn, durra, imphee, jowar, milo	Annual	H	3
	*Triticum aestivum* L.	Common wheat, bread wheat, wheat	Annual	H	4
	*Triticum dicoccon* (Schrank) Schübl.	Farro, emmer wheat	Annual	H	3
	*Triticum spelta* L.	Spelt	Annual	H	4
	*Triticum turgidum subsp. turanicum (Jakubz.) Á. Löve & D. Löve*	Kamut	Annual	H	2
	*Zea mays* L.	Maize, sweet corn, corn	Annual	H	4
	*Zizania* sp. L.	Wild rice			
*Polygonaceae*	*Fagopyrum esculentum* Moench	Buckwheat	Annual	H	4
*Proteaceae*	*Macadamia* sp. F.Muell.	Macadamia			
*Rosaceae*	*Fragaria × ananasa* Duchesne	Strawberry	Perennial	H	5
	*Malus domestica* Borkh.	Apple	Perennial	T	5
	*Prunus amygdalus* Batsch	Almond	Perennial	S or T	5
	*Prunus armeniaca* L.	Apricot	Perennial	T	5
	*Prunus domestica* L.	Prune, plume	Perennial	S or T	5
	*Rubus idaeus* L.	Raspberry, red raspberry	Perennial	S	5
	*Rubus × loganobaccus* L.H. Bailey	Loganberry	Perennial	S	4
*Rubiaceae*	*Gardenia* sp. J.Ellis	Gardenia			
	*Hydnophytum papuanum* Becc.	Maze, ant plant, ant house plant			
	*Coffea canephora* Pierre ex A.Froehner	Coffea, coffee	Perennial	S or T	3
*Rutaceae*	*Citrus* sp. L.	Citrus			
	*Citrus × tangerina* Tanaka	Tangerine	Perennial	T	4
*Santalaceae*	*Santalum yasi* Bertero	Ahi, yasi	Perennial	S or T	None known
*Sapindaceae*	*Acer* sp. L.	Maple			
*Sapotaceae*	*Sideroxylon spinosum* L.	Argan tree	Perennial	T	4
	*Vitellaria paradoxa* C.F.Gaertn.	Shea, shea butter tree, shea tree	Perennial	T	4
*Simmondsiaceae*	*Simmondsia chinensis* (Link) C.K. Schneid.	Jojoba	Perennial	S	2
*Solanaceae*	*Nicotiana* L.	Tobacco			
	*Capsicum annuum* L.	Paprika, pepper	Annual or perennial	H	4
	*Solanum lycopersicum* L.	Tomato	Annual	H	5
	*Solanum tuberosum* L.	Potato	Perennial	H	5
*Theaceae*	*Camellia sinensis* (L.) Kuntze	Tea, green tea, camellia	Perennial	S or T	5
*Vitaceae*	*Vitis vinifera* L.	Grape	Perennial	H	5
*Zingiberaceae*	*Curcuma longa* L.	Turmeric	Perennial	H	3

**Table A4:** The list of plants and their constituents that might be used in plant-based meat analogues (PBMA) according to reviewed patents developed in the years 2014–2023.

Family	Latin Name of Taxon	Common Name(s) of Taxon	Constituent	Number of Inventions	References in Chronological Order
*Actinidiaceae*	*Actinidia* sp. Lindl.	Kiwifruit	Lipids	1	[52]
			Proteins	1	[194]
*Adoxaceae*	*Sambucus* sp. L.	Elder, elderflower, elderberry	Polyphenols	1	[162]
*Amaranthaceae*	*Amaranthus* sp. L.	Amaranth	Lipids	2	[52,200]
			Polyphenols	1	[105]
			Proteins	6	[153,164,174,176,196,212]
	*Beta vulgaris L.*	Beet, beetroot	Fibre	2	[49,144]
			Polyphenols	11	[91,104,105,137,138,139,162,163,169,171,189]
	*Beta vulgaris L. subsp. vulgaris*	Sugar beet	Fibre	2	[94,144]
			Proteins	1	[50]
	*Celosia* sp. L.	Cockscomb	Proteins	1	[153]
	*Chenopodium pallidicaule* Aellen	Kaniwa, cañihua	Proteins	1	[153]
	*Chenopodium quinoa* Willd.	Quinoa	Fibre	1	[135]
			Lipids	2	[52,200]
			Proteins	20	[52,74,78,85,92,103,108,115,116,147,148,153,159,164,166,174,176,196,202,212]
			Starch	1	[135],
	*Spinacia oleracea* L.	Spinach	Fiber	1	[128]
			Polyphenols	1	[131]
			Proteins	2	[78,164]
*Amaryllidaceae*	*Allium cepa* L.	Onion	Lipids	1	[170]
*Anacardiaceae*	*Anacardium occidentale* L.	Cashew	Lipids	4	[52,80,182,200]
			Proteins	5	[116,191,194,218,219]
	*Mangifera indica* L.	Mango	Lipids	9	[49,50,120,121,122,161,196,204,205]
	*Pistacia vera* L.	Pistachi, pistachio	Lipids	3	[80,200]
			Proteins	3	[194,218,219]
*Annonaceae*	*Annona cherimola Mill.*	Cherimoya, chirimuya	Proteins	1	[164]
*Apiaceae*	*Apium graveolens* L.	Celery, Wild celery	Fiber	1	[49]
			Proteins	1	[78]
	*Arracacia xanthorrhiza* Bancr.	Arracacha, racacha	Starch	1	[200]
	*Coriandrum sativum* L.	Coriander	Lipids	3	[52,182,200]
	*Daucus carota* subsp*. sativus (*Hoffm.) Schübl. & G. Martens	Carrot	Fibre	6	[49,69,104,105,201,208]
			Lipids	1	[211]
			Polyphenols	7	[91,105,139,162,163,171,211]
			Proteins	1	[78]
	*Petroselinum crispim (*Mill.) Fuss	Parsley, garden parsley	Fibre	1	[49]
*Araceae*	*Amorphophallus konjac* K. Koch	Konjac, koniak, Devil’s tongue	Fibre	1	[195]
			Gum	14	[123,146,157,161,165,176,178,183,196,206,208,212,213,220]
			Starch	3	[200,203,210]
	*Colocasia esculenta (*L.) Schott	Taro	Fibre	1	[128]
			Proteins	1	[212]
			Starch	4	[44,79,157,200]
*Arecaceae*	*Attalea speciosa* Mart.	Babassu	Lipids	9	[49,50,52,81,121,196,200,205,212]
			Proteins	1	[212]
	*Cocos nucifera* L.	Coconut	Fibre	1	[105]
			Lipids	60	[42,49,50,52,53,54,58,68,74,75,80,85,86,88,92,96,97,100,103,104,114,116,120,121,122,134,139,140,141,142,145,148,150,151,159,162,163,165,166,168,170,171,172,173,179,181,182,191,194,196,200,201,202,207,208,212,213,218,219]
			Proteins	6	[42,48,168,194,218,219]
	*Euterpe oleracea* Mart.	Acai	Lipids	3	[52,80,200]
*Asparagaceae*	*Agave sisalana* Perrine	Sisal	Fibre	1	[116]
	*Asparagus officinalis* L.	Asparagus	Proteins	3	[164,218,219]
*Asteraceae*	*Artemisia vulgaris* L.	Mugwort	Polyphenols	1	[131]
	*Calendula officinalis* L.	Calendula, Pot marigold	Lipids	1	[182]
	*Carthamus tinctorium* L.	Safflower, benibana	Lipids	39	[42,44,48,49,50,52,53,54,65,74,79,80,81,92,99,108,111,120,121,122,139,140,145,157,158,162,163,166,168,172,178,182,191,196,200,205,207,208,212]
			Polyphenols	3	[56,162,189]
			Proteins	6	[42,48,168,194,218,219]
	*Cynara cardunculus* var*. scolymus* L.	Artichoke, Globe artichoke	Lipids	1	[52]
			Proteins	4	[164,194,218,219]
	*Guizotia abyssinica* (L. f.) Cass	Ramtil, Niger seed	Lipids	2	[52,200]
	*Helianthus annuus* L.	Sunflower	Fibre	1	[102]
			Lipids	72	[42,44,48,49,50,52,53,54,56,58,65,74,75,77,78,79,80,81,92,97,98,99,105,108,111,114,115,116,120,121,122,123,128,130,135,139,140,142,145,148,151,152,153,154,157,158,159,162,163,166,167,168,169,170,171,172,173,178,182,189,190,191,196,200,202,203,205,207,208,209,212,215]
			Proteins	34	[42,43,48,51,58,62,64,75,80,116,117,121,122,124,133,139,159,164,165,167,168,170,174,175,176,178,191,194,196,213,215,218,219,220]
	*Tussilago farfara* L.	Coltsfoot	Fibre	1	[128]
*Betulaceae*	*Corylus avellana* L.	Hazelnut, hazel	Lipids	10	[44,52,53,80,140,162,182,191,200,207]
			Proteins	5	[191,194,202,218,219]
*Bixaceae*	*Bixa orellana* L.	Annatto	Polyphenols	2	[139,162]
*Boraginaceae*	*Borago officinalis* L.	Borago, Borage, starflower	Lipids	6	[52,121,196,200,205,212]
*Brassicaceae*	*Brassica carinata* A.Braun	Carinata, Ethiopian rape, Ethiopian mustard, Abyssinian Cabbage	Lipids	1	[178]
	*Brassica juncea (*L.) Czern.	Brown mustard, Chinese mustard, Indian mustard, Korean green mustard, leaf mustard, oriental mustard, vegetable mustard	Proteins	1	[90]
	*Brassica napus* L.	Canola, rapeseed, rape, colza	Lipids	76	[42,44,47,48,49,50,52,53,54,56,58,60,65,74,76,78,79,80,81,82,89,92,95,96,97,99,104,105,108,111,113,114,116,119,120,121,122,123,130,137,139,140,141,142,143,145,148,152,154,157,158,159,162,166,167,168,171,172,173,178,179,182,189,190,191,194,195,196,200,202,204,205,207,208,212,219]
			Proteins	42	[42,48,50,51,53,56,58,60,62,64,75,78,82,90,99,102,117,121,133,140,142,158,159,160,161,163,164,167,168,172,175,176,178,194,195,196,198,210,214,218,219,220]
	*Brassica oleracea* L.	Kale, leaf cabbage, wild cabbage	Fibre	1	[49]
			Polyphenols	3	[91,162,163]
			Proteins	3	[90,164,202]
	*Brassica oleracea* var*. italica* Plenck	Broccoli	Fiber	1	[49]
			Proteins	6	[90,164,194,202,218,219]
	*Brassica oleracea* var*. botrytis* L.	Cauliflower	Proteins	4	[90,194,218,219]
	*Brassica oleracea* var*. gongylodes* L.	Kohlarbi	Proteins	1	[90]
	*Brassica rapa* (L.) Koch.	Field mustard, turnip	Lipids	1	[96]
	*Camelina sativa* (L.) Crantz	False flax, camelina, gold of pleasure, wild flax, linseed dodder, German sesame, Siberian oilseed	Lipids	20	[49,50,65,80,99,111,116,121,139,152,158,163,172,178,182,191,196,200,208,212]
			Proteins	8	[50,62,116,159,164,194,218,219]
	*Moringa oleifera* Lam.	Horseradish Tree, moringa	Lipids	1	[168]
	*Raphanus sativus* L.	Radish	Fibre	1	[128]
			Polyphenols	5	[56,104,105,139,162]
	*Rhamphospermum nigrum* L. Al-Shehbaz	Black mustard	Proteins	1	[90]
	*Sinapis alba* L.	White mustard	Proteins	1	[90]
*Bromeliaceae*	*Ananas comosus* (*L.) Merr.*	Pineapple	Fibre	1	[116]
*Campanulaceae*	*Codonopsis lanceolata(*Siebold & Zucc.) Trautv.	Deodeok, todok, lance asiabell	Fibre	1	[128]
*Cannabaceae*	*Cannabis sativa* L.	Hemp	Fibre	1	[128]
			Lipids	4	[52,80,182,200]
			Proteins	20	[52,62,78,85,103,105,133,140,147,148,159,164,166,175,176,190,191,197,202,205]
*Caryocaraceae*	*Caryocar brasiliense* Cambess.	Pequi	Lipids	2	[52,200]
*Clusiaceae*	*Allanblackia floribunda* Oliv.	Tallow tree, allablackia, vegetable tallow	Lipids	2	[54,161]
	*Garcinia indica* Choisy	Kokum, goa butter	Lipids	2	[81,161]
*Combretaceae*	*Terminalia anogeissiana* Gere & Boatwr.	Axlewood, bakli, baajhi, dhau, dhawa, dhawra, dhaora	Gum	1	[139]
*Convolvulaceae*	*Ipomoea batatas* (L.) Lam.	Sweet potato	Fibre	5	[49,69,105,128,135]
			Polyphenols	2	[84,162],
			Proteins	6	[105,164,190,194,212,219]
			Starch	12	[44,53,65,79,89,140,157,166,186,199,200,221]
*Cucurbitaceae*	*Citrullus lanatus* (Thunb.) Matsum. & Nakai	Water melon	Lipids	1	[52]
	*Cucurbita foetidissima* Kunth	Buffalo gourd	Lipids	2	[52,200]
	*Cucurbita pepo* L.	Pumpkin	Fibre	2	[69,128]
			Lipids	5	[52,182,189,191,200]
			Polyphenols	1	[131],
			Proteins	9	[62,82,116,124,170,175,176,191,197]
	*C. pepo var. cylindrica* L.	Zucchini	Fibre	1	[49]
*Dipterocarpaceae*	*Shorea robusta* Roth	sal tree, sāla, shala, sakhua, sarai	Lipids	1	[161]
	*Shorea stenoptera* Burck.	Borneo tallow tree, illipe	Lipids	4	[52,161,200,202]
*Ericaceae*	*Oxycoccus* sp. Hill.	Cranberry	Polyphenols	1	[162]
			Proteins	2	[83,174]
	*Vaccinium* sect*. cyanococcus* Rydb.	Blueberry	Polyphenols	2	[162,189]
*Euphorbiaceae*	*Jatropha* sp. L.	Jatropha	Lipids	1	[182]
	*Manihot esculenta* Crantz.	Tapioca, cassava	Fibre	3	[69,105,208]
			Proteins	6	[90,116,194,196,212,219]
			Starch	20	[44,53,65,75,79,87,89,102,104,130,140,157,166,169,186,199,200,208,210,221]
	*Plukenetia volubilis* L.	Sacha ichni	Proteins	1	[174]
	*Ricinus communis* L.	Castor, castor bean, castor-oil plant	Lipids	3	[182,189,200]
	*Vernicia fordii* (Hemsl.) Airy Shaw	Tung tree, tungoil tree, kalo nut tree, China wood-oil tree	Lipids	1	[182]
*Fabaceae*	*Acacia* sp. Mill.	Wattle	Gum	3	[50,139,141]
	*Arachis hypogaea* L.	Peanut, groundnut, goober, goober pea, pindar, monkey nut, arachis	Lipids	43	[44,49,50,52,53,56,65,68,74,80,89,92,97,105,108,116,119,120,121,139,140,142,145,148,152,158,162,166,168,172,178,180,182,189,190,191,196,200,202,205,207,208,212]
			Proteins	29	[42,46,48,52,57,62,78,79,93,96,99,102,116,153,159,164,166,168,170,174,180,191,194,196,200,202,212,214,219]
	*Astragalus gummifer* Labill.	tragacanth, gum tragacanth milkvetch	Gum	10	[80,81,115,120,139,142,178,194,202,220]
	*Cajanus cajan* (L.) Millsp	Pigeon pea	Fibre	1	[135]
			Proteins	2	[52,153]
			Starch	1	[135]
	*Cassia* sp. L.	Cassia	Gum	1	[157]
	*Ceratonia siliqua* L.	Carob, locust bean	Fibre	1	[195]
			Gum	23	[50,75,76,80,81,87,115,120,139,142,145,146,157,165,178,183,193,194,199,202,208,213,220]
			Lipids	2	[52,200]
			Proteins	3	[52,153,166]
			Starch	1	[200]
	*Cicer arietinum* L.	Chickpea, channa, chana, garbanzo	Fibre	2	[69,135]
			Proteins	60	[42,50,51,52,55,57,62,64,70,72,74,76,80,81,85,92,96,97,102,103,105,114,115,116,117,125,135,142,148,151,152,153,154,155,159,163,164,166,167,168,173,174,176,178,190,191,194,196,197,200,201,202,206,210,212,214,216,219,220,221]
			Starch	3	[53,135,140]
	*Cyamopsis tetragonoloba* (L.) Taub	Guar, cluster bean	Gum	24	[50,75,76,80,87,104,115,120,123,139,142,146,153,165,178,183,189,194,199,202,208,213,220,221]
			Proteins	1	[114]
			Starch	1	[200]
	*Glycine max* L. Merr.	Soybean	Fibre	1	[105]
			Lipids	70	[42,44,48,49,50,52,53,54,56,58,60,61,65,74,78,79,80,81,89,92,97,99,105,108,109,111,116,119,120,121,122,123,130,137,139,140,142,143,145,148,152,153,154,157,158,162,163,166,168,170,171,172,173,178,182,189,190,191,196,200,204,205,207,208,209,210,212,217,218,219]
			Proteins	139	[39,40,41,42,43,44,48,49,50,51,52,53,54,56,57,58,59,60,61,62,64,65,67,68,70,71,73,74,75,76,77,79,80,81,82,84,85,86,88,89,90,93,94,95,97,98,99,100,102,103,104,105,108,109,110,111,112,113,115,116,117,118,119,120,121,122,124,126,128,131,132,133,135,136,139,140,142,143,144,148,149,151,152,153,154,155,156,157,158,159,160,162,163,164,165,166,167,168,169,171,172,173,174,176,177,178,180,181,183,184,185,186,187,188,189,190,191,192,194,195,196,197,198,199,200,201,202,206,207,209,210,213,214,215,217,218,219,220,221]
	*Lablab purpureus* (L.) Sweet	Hyacinth Bean	Proteins	1	[114]
	*Lathyrus oleraceus* Lam.	Pea	Fibre	12	[49,87,102,105,116,135,141,161,178,182,208,214]
			Proteins	118	[39,42,43,46,47,48,50,51,52,53,54,56,57,59,60,62,65,72,74,75,76,77,79,80,81,82,84,85,87,90,92,94,95,96,97,98,99,100,102,103,104,105,106,107,108,111,114,115,116,117,118,120,121,122,124,125,126,127,133,135,136,139,140,141,142,144,148,151,152,154,155,156,157,159,160,162,163,164,165,166,167,168,169,170,171,173,174,176,178,181,182,183,185,186,188,190,191,192,194,195,196,197,198,200,201,202,204,207,208,210,212,213,214,215,216,219,220,221]
			Starch	14	[53,65,75,102,118,129,135,140,146,153,159,182,208,210]
	*Lupinus* sp. L.	Lupine, lupin	Fibre	4	[45,102,135,214]
			Lipids	1	[182]
			Proteins	43	[43,47,50,51,52,57,58,62,64,77,78,81,85,94,100,103,111,114,116,117,122,142,151,153,155,158,160,161,162,163,164,166,167,174,176,178,196,204,207,212,214,216,220]
	*Medicago sativa* L.	Alfalfa	Fibre	1	[49]
			Lipids	1	[202]
			Proteins	13	[43,50,51,52,57,78,81,96,117,153,159,166,204]
	*Phaseolus lunatus* L.	Lima bean	Proteins	3	[114,191,212]
	*Phaseolus vulgaris* L.	Kidney bean, French bean	Fibre	1	[49]
			Proteins	34	[43,50,51,52,57,62,66,72,74,76,80,81,86,92,96,102,106,114,115,117,125,142,151,155,156,163,166,167,178,191,212,214,220,221]
			Starch	1	[129]
	*Prosopis* sp. L.	Mesquite	Proteins	3	[52,153,166]
	*Pueraria montana (*Lour.) Merr.	Kudzu, mealy kudzu	Starch	1	[200]
	*Tamarindus indica* L.	Tamarind	Proteins	1	[153]
	*Tara* sp. Molina	Tara	Gum	5	[81,115,161,199,220]
	*Trifolium* sp. L.	Clover	Proteins	5	[52,78,153,159,166]
	*Trigonella foenum-graecum* L.	Fenugreek	Fibre	1	[69]
			Gum	3	[81,161,220]
	*Vicia* sp.	Vetch	Proteins	1	[153]
	*Vicia faba* L.	Fava bean, broad bean, horse bean	Fibre	3	[102,135,214]
			Proteins	38	[46,52,57,62,74,80,85,90,96,99,102,103,105,111,114,116,133,141,153,160,161,163,164,174,176,178,191,194,195,197,198,200,210,214,216,219,220,221]
			Starch	2	[129,135]
	*Vicia lens* (L.) Coss. & Germ.	Lentil	Fibre	1	[135]
			Proteins	55	[43,51,52,57,62,70,72,74,77,78,85,92,97,100,102,103,105,106,108,111,114,116,118,122,125,135,139,148,155,159,160,161,162,163,164,166,168,174,176,178,190,191,194,196,197,198,202,207,210,212,214,219,220,221]
			Starch	3	[53,135,140]
	*Vigna angularis* (Willd.) Ohwi & H. Ohashi	Red bean, adzuki bean	Proteins	4	[102,194,214,219]
	*Vigna radiata* (L.) R. Wilczek	Mung bean	Fibre	2	[105,135]
			Proteins	31	[50,62,75,80,81,86,96,102,105,114,116,117,122,133,156,159,164,166,167,168,178,190,191,194,196,197,213,214,216,219,220]
			Starch	5	[53,65,135,140,199]
	*Vigna subterranea* (L.) Verdc.	Bambara bean	Proteins	1	[153]
	*Vigna unguiculata* subsp*. stenophylla* (Harv.) Maréchal, Mascherpa & Stainier	Cowpea	Proteins	9	[50,52,57,62,114,153,166,174,212]
	*Vigna unguiculata subsp. unguiculata* (L.) Walp.	Black-eyed bean, Black-eyed pea	Proteins	1	[72]
*Fagaceae*	*Fagus* sp. L.	Beech	Lipids	1	[80]
*Grossulariaceae*	*Ribes nigrum* L.	Blackcurrant	Lipids	8	[49,52,121,162,196,200,205,212]
			Polyphenols	3	[91,162,189]
	*Ribes rubrum*	Redcurrant	Polyphenols	1	[162]
	*Ribes uva-crispa* L.	Gooseberry	Polyphenols	1	[189]
			Proteins	1	[212]
*Juglandaceae*	*Carya illinoinensis* (Wangenh.) K.Koch	Pecan	Lipids	5	[52,80,182,191,200]
			Proteins	1	[191]
	*Juglans regia* L.	Walnut	Lipids	17	[49,50,52,65,74,80,81,99,120,121,162,191,196,200,205,207,212]
			Proteins	3	[116,191,202]
*Lamiaceae*	*Lallemantia royleana* Benth. in Wall.	Lallemantia, balangu	Lipids	2	[52,200]
	*Perilla frutescens* (L.) Britton	Perilla, egoma, shiso	Lipids	5	[52,65,128,172,200]
	*Salvia hispanica* L.	Mexican chia, chia	Proteins	12	[85,103,116,133,148,153,159,164,166,174,191,202]
*Lauraceae*	*Persea americana* Mill.	Avocado, avocado pear	Lipids	14	[52,54,97,104,139,142,145,162,166,170,182,191,200,207]
			Proteins	3	[194,218,219]
*Lecythidaceae*	*Bertholletia excelsa* Humb. &Bonpl.	Brasil nut, Brazil nut	Lipids	1	[182]
			Proteins	2	[191]
*Liliaceae*	*Erythronium japonicum* Decne.	Katakuri	Starch	1	[50]
*Limnanthaceae*	*Limnanthes* sp. R.Br.	Meadowfoam	Lipids	2	[52,200]
*Linaceae*	*Linum usitatissimum* L.	Flax, common flax, lineseed	Fibre	1	[208]
			Lipids	8	[52,81,120,122,145,166,191,205]
			Proteins	1	[191]
*Lythraceae*	*Punica granatum* L.	Pomegranate	Polyphenols	3	[78,91,162]
	*Abelmoschus esculentus* (L.) Moench	Okra	Lipids	1	[200]
	*Ceiba pentandra* (L.)Gaertn.	Kapok, kapok tree	Lipids	3	[52,168,200]
	*Gossypium* sp. L.	Cotton	Fibre	1	[214]
			Lipids	41	[44,49,50,52,53,54,58,65,80,81,89,92,108,111,116,120,121,122,123,139,140,145,148,158,162,163,166,168,172,178,182,189,191,196,200,202,204,205,207,208,212]
			Proteins	14	[42,48,50,58,64,79,99,159,165,168,170,194,218,219]
	*Hibiscus* sp. L.	Hibiscus	Polyphenols	2	[139,162]
	*Hibiscus cannabinus* L.	Kenaf	Lipids	1	[182]
	*Malva* sp. L.	Mallow	Fibre	1	[128]
	*Sterculia urens* Roxb.	Kulu, Indian tragacanth, karaya, gum karaya, katira, sterculia gum, kateera gum	Gum	4	[157,161,178,202]
	*Theobroma cacao* L.	Cocoa, cacao	Lipids	19	[42,49,50,56,96,105,120,121,123,161,166,182,190,194,196,201,202,205,215]
			Polyphenols	1	[138]
			Proteins	2	[52,166]
*Marantaceae*	*Maranta arundinacea* L.	Arrowroot, maranta, West Indian arrowroot, obedience plant, Bermuda arrowroot, araru, araruta, Ararat, hulankeeriya	Proteins	1	[196]
			Starch	9	[44,50,53,79,120,140,157,199,200]
*Moraceae*	*Artocarpus altilis* (Parkinson) Fosberg	Breadfruit	Starch	3	[44,79,157]
	*Artocarpus camansi* Blanco	Breadnut	Proteins	1	[153]
	*Artocarpus heterophyllus* Lam.	Jackfruit	Fibre	1	[169]
			Proteins	3	[194,218,219]
	*Morus* sp. L.	Mulberry	Proteins	1	[164]
*Musaceae*	*Musa* sp.	Banana	Fibre	3	[105,116,169]
			Proteins	4	[164,194,218,219]
	*Musa textilis* Née	Abaca	Fibre	1	[116]
			Starch	4	[44,79,157,200]
*Myrtaceae*	*Psidium guajava* L.	Common guava, yellow guava, lemon guava, apple guana, guava	Proteins	1	[164]
*Nelumbonaceae*	*Nelumbo nucifera* Gaertn.	Lotus, Indian lotus, sacred water lotus	Starch	1	[199]
*Oleaceae*	*Olea europaea* L.	Olive	Lipids	59	[44,49,50,52,53,54,58,60,65,74,79,80,81,89,96,97,99,105,108,111,114,116,119,120,121,122,128,139,140,142,145,148,157,158,162,163,166,167,168,170,171,172,178,179,182,189,191,194,196,200,202,204,205,207,208,209,210,212,219]
			Proteins	3	[194,218,219]
*Onagraceae*	*Oenothera biennis* L.	Evening primose	Lipids	4	[52,108,168,200]
*Oxalidaceae*	*Oxalis tuberosa* Molina	Oca, uqa, yam	Fibre	1	[69]
			Proteins	2	[116,212]
			Starch	5	[44,53,79,157,200]
*Papaveraceae*	*Papaver* sp. L.	Poppy	Lipids	3	[52,80,200]
*Pedaliaceae*	*Sesamum indicum* L.	Sesame	Lipids	28	[42,50,52,65,80,81,89,105,108,116,120,121,128,139,142,162,168,170,172,182,190,191,196,200,205,207,208,212]
			Proteins	18	[42,48,50,58,62,64,105,116,139,159,168,174,190,191,194,202,218,219]
*Pinaceae*	*Pinus sp.* L.	Pine	Lipids	2	[52,200]
			Proteins	1	[191]
*Plantaginaceae*	*Plantago major* L.	Broadleaf plantain, common plantain, white man’s footprint, waybread, greater plantain	Starch	3	[44,79,157]
	*Plantago ovata* Forsk	Psyllium, blond psyllium	Fibre	9	[49,69,105,125,146,195,206,208,213]
*Poaceae*	*Avena sativa* L.	Oats, common oat	Fibre	7	[49,94,102,135,143,208,214]
			Lipids	3	[182,191,202]
			Proteins	40	[43,46,51,52,58,59,62,64,70,74,78,80,92,96,105,113,116,122,133,135,148,154,159,164,165,166,168,174,176,178,190,191,194,196,197,202,212,218,219,220]
			Starch	6	[46,66,71,135,153,182]
	*Cenchrus americanus* (L.) Morrone	Millet, pearl millet	Fibre	1	[208]
			Proteins	7	[52,66,78,164,166,196,212]
	*Digitariaexilis*(Kippist) Stapf	Fonio, fonio millet	Proteins	2	[52,166]
			Lipids	1	[102]
	*Eragrostis tef* (Zucc.) Trotter	Teff, tef	Proteins	2	[174,212]
	*Hordeum vulgare* L.	Barley	Fibre	2	[135,208]
			Lipids	1	[202]
			Proteins	25	[42,43,50,51,52,53,62,66,67,74,78,92,96,108,140,153,154,166,168,176,191,194,196,218,219]
			Starch	8	[44,53,79,87,135,140,157,200]
	*Oryza sativa* L.	Rice	Fibre	3	[102,135,214]
			Lipids	27	[42,49,50,52,80,81,97,105,108,120,122,130,139,143,145,158,162,168,170,182,190,194,200,207,208,210,219]
			Proteins	46	[39,42,44,48,50,52,53,60,62,66,74,75,77,78,79,96,97,99,104,111,113,115,116,117,124,133,140,148,154,161,166,167,168,175,178,185,191,194,196,198,201,212,216,218,219,220]
			Starch	17	[39,44,53,65,71,79,87,135,140,153,157,166,186,199,200,208,221]
	*Secale cereale* L.	Rye	Fibre	1	[135]
			Proteins	11	[52,62,66,74,78,92,96,116,166,196,215]
			Starch	1	[135]
	*Saccharum* sp. L.	Sugar cane, sugarcane	Fibre	2	[94,208]
	*Sorghum bicolor* L. Moench	Sorghum, great millet, broomcorn, guinea corn, durra, imphee, jowar, milo	Fibre	1	[135]
			Lipids	1	[202]
			Proteins	6	[52,78,115,166,174,196]
			Starch	8	[44,53,79,135,140,157,166,200]
	*Triticum aestivum* L.	Common wheat, bread wheat, wheat	Fibre	6	[49,94,135,143,170,208]
			Lipids	10	[50,80,81,120,121,145,196,200,205,212]
			Proteins	89	[39,42,43,44,48,49,50,52,53,56,58,59,62,64,65,66,67,70,74,75,77,78,79,80,82,85,92,93,97,98,99,100,102,103,105,107,108,109,111,113,116,122,124,126,128,133,135,136,140,144,148,151,152,157,159,160,162,163,165,166,169,170,171,173,174,175,176,178,181,183,184,190,191,193,194,196,197,198,199,200,206,207,208,210,212,215,218,219,220]
			Starch	23	[44,53,54,65,79,87,89,92,98,102,130,135,140,148,153,157,166,186,199,200,208,216,221]
	*Triticum dicoccon (*Schrank) Schübl.	Farro, emmer wheat	Proteins	1	[153]
	*Triticum spelta* L.	Spelt	Proteins	6	[105,148,164,190,191,212]
	*Triticum turgidum* subsp*. turanicum* (Jakubz.) Á.Löve	Kamut	Proteins	1	[196]
	*Zea mays* L.	Maize, sweet corn, corn	Fibre	5	[49,102,135,208,214]
			Lipids	59	[42,44,48,49,50,52,53,54,56,58,60,65,66,74,79,80,81,86,89,99,104,105,108,111,114,116,119,120,121,122,139,140,142,143,145,152,157,158,162,163,166,168,170,171,172,178,182,189,190,191,194,196,202,204,205,207,208,212,219]
			Proteins	39	[42,43,50,51,52,53,62,74,78,79,92,96,97,99,115,116,117,140,144,159,164,165,166,167,168,170,173,174,175,176,191,194,196,197,200,210,212,218,219]
			Starch	34	[44,50,53,54,58,63,65,67,74,75,79,87,89,101,102,104,118,120,130,135,137,139,140,157,159,165,166,186,193,203,207,208,210,221]
	*Zizania* sp. L.	Wild rice	Proteins	2	[50,164]
*Polygonaceae*	*Fagopyrum esculentum Moench*	Buckwheat	Fibre	1	[135]
			Proteins	10	[52,66,74,78,92,153,166,176,196,212]
			Starch	2	[135,200]
*Proteaceae*	*Macadamia* sp. F.Muell.	Macadamia	Lipids	10	[49,52,80,121,128,145,182,196,200,205]
			Proteins	3	[194,218,219]
*Rosaceae*	*Fragaria × ananasa* Duchesne	Strawberry	Polyphenols	3	[162,163,189]
	*Hippophae rhamnoides* L.	Sea buckhorn	Lipids	5	[49,121,196,205,212]
	*Malus domestica* Borkh.	Apple	Fibre	8	[49,69,102,116,135,169,208,214]
			Polyphenols	1	[162]
	*Prunus amygdalus* Batsch	Almond	Lipids	19	[44,50,52,53,80,81,120,121,139,140,145,162,191,196,200,204,205,207,212]
			Proteins	11	[52,78,116,164,166,170,191,194,200,202,219]
	*Prunus armeniaca* L.	Apricot	Lipids	2	[52,200]
	*Prunus domestica* L.	Prune, plume	Lipids	1	[200]
	*Rubus idaeus* L.	Raspberry, red raspberry	Polyphenols	3	[162,163,189]
	*Rubus × loganobaccus* L.H. Bailey	Loganberry	Polyphenols	1	[189]
*Rubiaceae*	*Gardenia* sp. J.Ellis	Gardenia	Polyphenols	2	[131,138]
	*Hydnophytum papuanum* Becc.	Maze, ant plant, ant house plant	Lipids	1	[202]
	*Coffea canephora* Pierre ex A.Froehner	Coffea, coffee	Lipids	1	[182]
			Polyphenols	1	[162]
*Rutaceae*	*Citrus* sp. L.	Citrus	Fibre	2	[144]
	*Citrus × tangerina* Tanaka	Tangerine	Lipids	1	[128]
*Santalaceae*	*Santalum yasi* Bertero	Ahi, yasi	Lipids	1	[205]
*Sapindaceae*	*Acer* sp. L.	Maple	Fibre	1	[125]
*Sapotaceae*	*Sideroxylon spinosum* L.	Argan tree	Lipids	2	[52,200]
	*Vitellaria paradowa* C.F.Gaertn.	Shea, shea butter tree, Shea tree	Lipids	22	[49,50,56,75,80,81,85,100,103,104,105,108,120,121,123,150,190,191,194,196,201,205]
*Simmondsiaceae*	*Simmondsia chinensis* (Link) C.K. Schneid.	Jojoba	Lipids	2	[145,182]
*Solanaceae*	*Nicotiana* sp. L.	Tobacco	Proteins	1	[50]
	*Capsicum annuum* L.	Paprika, pepper	Polyphenols	2	[105,162]
	*Solanum lycopersicum* L.	Tomato	Fibre	1	[128]
			Polyphenols	4	[104,105,139,162]
	*Solanum tuberosum* L.	Potato	Fibre	10	[49,69,102,105,121,135,141,208,213,214]
			Polyphenols	2	[162,171]
			Proteins	44	[43,51,52,58,64,69,78,80,82,90,94,95,97,104,105,115,116,117,122,133,140,142,144,146,148,150,151,161,164,166,167,170,173,175,176,178,194,195,196,202,212,216,219,220]
			Starch	31	[44,50,53,54,65,74,75,79,87,89,104,107,109,120,130,137,140,153,157,159,165,166,181,186,199,200,201,208,210,219,221]
*Theaceae*	*Camellia sinensis* (L.) Kuntze	Tea, Green tea, camellia	Lipids	3	[52,182,200]
			Polyphenols	3	[67,78,162]
*Vitaceae*	*Vitis vinifera* L.	Grape	Lipids	14	[44,52,65,80,97,105,139,142,145,182,190,200,202,210]
			Polyphenols	1	[162]
*Zingiberaceae*	*Curcuma longa* L.	Turmeric	Polyphenols	1	[131]
